# An international survey on the clinical use of rigid and deformable image registration in radiotherapy

**DOI:** 10.1002/acm2.12957

**Published:** 2020-09-11

**Authors:** Johnson Yuen, Jeffrey Barber, Anna Ralston, Alison Gray, Amy Walker, Nicholas Hardcastle, Laurel Schmidt, Kristie Harrison, Joel Poder, Jonathan R. Sykes, Michael G. Jameson

**Affiliations:** ^1^ St George Hospital Cancer Care Centre Kogarah NSW 2217 Australia; ^2^ Liverpool and Macarthur Cancer Therapy Centres Campbelltown NSW Australia; ^3^ South Western Clinical School University of New South Wales Sydney Australia; ^4^ Ingham Institute for Applied Medical Research Sydney Australia; ^5^ Centre for Medical Radiation Physics University of Wollongong Wollongong NSW Australia; ^6^ Physical Sciences Peter MacCallum Cancer Centre Melbourne Australia; ^7^ GenesisCare Newcastle NSW Australia; ^8^ Blacktown Cancer & Hematology Centre Sydney West Cancer Network Sydney Australia; ^9^ Institute of Medical Physics University of Sydney Sydney Australia; ^10^ Present address: GenesisCare Sydney NSW Australia

**Keywords:** deformable, image registration, rigid, survey

## Abstract

**Objectives:**

Rigid image registration (RIR) and deformable image registration (DIR) are widely used in radiotherapy. This project aims to capture current international approaches to image registration.

**Methods:**

A survey was designed to identify variations in use, resources, implementation, and decision‐making criteria for clinical image registration. This was distributed to radiotherapy centers internationally in 2018.

**Results:**

There were 57 responses internationally, from the Americas (46%), Australia/New Zealand (32%), Europe (12%), and Asia (10%). Rigid image registration and DIR were used clinically for computed tomography (CT)‐CT registration (96% and 51%, respectively), followed by CT‐PET (81% and 47%), CT‐CBCT (84% and 19%), CT‐MR (93% and 19%), MR‐MR (49% and 5%), and CT‐US (9% and 0%). Respondent centers performed DIR using dedicated software (75%) and treatment planning systems (29%), with 84% having some form of DIR software. Centers have clinically implemented DIR for atlas‐based segmentation (47%), multi‐modality treatment planning (65%), and dose deformation (63%). The clinical use of DIR for multi‐modality treatment planning and accounting for retreatments was considered to have the highest benefit‐to‐risk ratio (69% and 67%, respectively).

**Conclusions:**

This survey data provides useful insights on where, when, and how image registration has been implemented in radiotherapy centers around the world. DIR is mainly in clinical use for CT‐CT (51%) and CT‐PET (47%) for the head and neck (43–57% over all use cases) region. The highest benefit‐risk ratio for clinical use of DIR was for multi‐modality treatment planning and accounting for retreatments, which also had higher clinical use than for adaptive radiotherapy and atlas‐based segmentation.

## 1 INTRODUCTION

Medical image registration (IR) enables a user to combine and compare information from multiple images and has applications in radiology,^1^ nuclear medicine,^2^, ^3^ and radiotherapy.^4^, ^5^, ^6^, ^7^ The use of images is increasing in healthcare,^1^ and applications of IR can benefit patients in diagnosis, planning, treatment, and response assessment.^1^ Almost every software system that uses images in radiotherapy has rigid image registration (RIR) functionalities^4^ that involves translational and rotational corrections with up to 6 degrees of freedom. More complex is deformable image registration (DIR) which has 3 degrees of freedom for every voxel in the image.^4^ Validation and clinical translation of IR have been considered challenging for over 20 yr,^8^ with DIR validation considered an unresolved subject.^5^, ^6^

The report of the AAPM Radiation Therapy Committee Task Group No. 132^4^ (TG 132) provides formal quality assurance guidelines for IR. This report increased awareness in the need for formal quality management to better understand, communicate, and manage the uncertainty of both rigid and deformable image registration. Accuracy in IR has direct and indirect implications for clinical risk, such as interpretation of PTV.^9^ Rigid image registration is a well‐established but limited technique when the size, shape, or the orientation of structure is different between the two scans. DIR can achieve superior spatial congruence between image pairs in certain conditions (such as high contrast regions), but can be considered *ill‐defined and over‐constrained*.^4^ As a single IR technique (RIR or DIR) may not be robust for all circumstances,^10^ there is value in data to help select an *appropriate* IR technique, and data to help decide on a *per‐patient* IR quality assurance.^4^

With image‐guided radiotherapy (IGRT) surveys emphasizing the growing importance of imaging,^11^, ^12^, ^13^, ^14^ there is also DIR adoption data^14^, ^15^, ^16^ that highlights the increase in complex IRs. Commercial solutions and clinical needs are factors driving DIR use despite published limitations and risks, resulting in a need to better understand when, what, who, and how IR are clinically used. The aim of the survey was thus to measure useful reference data for the development of productive implementation and quality assurance strategies adapted toward clinical resources and requirements.

## 2 **MATERIALS AND METHOD**S

### 2.A Question generation and review

The survey was created with Google Forms (Google LLC, California, US) and was exploratory in nature. The survey was not endorsed in an official capacity and it was not anticipated that all centers would respond. Survey questions were designed to assess significant variations in the status of implementation and clinical use of IR, concentrating not only on DIR but also targeting RIR.

There were two versions of the survey, both in English: (a) for radiotherapy centers in Australia/New Zealand (ANZ), and (b) for international radiotherapy centers. Differences in the surveys were that (a) ANZ respondents were questioned on which state/territory they were within, and (b) wording was changed because Dosimetrists as a staff speciality was not applicable in ANZ as Radiation Therapists (RTs) perform both treatment and treatment planning roles. Each department was requested to provide a single response from a multidisciplinary team of Medical Physicists, RTs, Dosimetrists, and Radiation Oncologists (ROs). There were 27 *standard survey* questions on core IR practice patterns, with expected completion within 10 min. There were 54 *extended survey* questions that focused on DIR implementation with an additional 30 min required (Appendix S1).

*Standard survey* questions included

- department characteristics
- RIR/DIR utilization by image modality pair
- DIR software utilized
- first planned or actual adoption of DIR for each use case
- use of IR request and report forms
- IR QA mechanisms
- IR training
- IR involvement by staff discipline
- IR challenges
- IR uncertainties.

*Extended survey* questions included

- ideal levels of staff involvement in DIR
- RIR/DIR applications by anatomical site
- process‐based evaluation of responsibilities
- process‐based evaluation of satisfaction levels
- metrics used for IR QA
- validation datasets used
- number of datasets by application
- patient specific QA by application
- IR techniques
- criteria for clinical release of IR
- benefit‐to‐risk for IR
- quality measures for safe use of RIR/DIR.

Table 9 contains the list of processes (xiii, xiv).

### 2.B Survey data collection and analysis

On the April 19, 2018, the ANZ survey was distributed through emails to Medical Physics Directors, while the INTL survey was distributed through the MEDPHYS email list hosted by Wayne State University.^16^ Additional survey responses were obtained by emailing AFOMP (Asia‐Oceania Federation of Organizations for Medical Physics) representatives from each region in Asia. The deadline was extended on the August 24, 2018 and closed on the September 1, 2018. Survey data were exported from Google Forms^TM^ (Google LLC, California, US) into Microsoft Excel^TM^ (Microsoft Corporation, Washington, US) for descriptive data analysis.

The data in the spreadsheet were anonymized. Search functions were used in Microsoft Excel^TM^ to parse responses into number values, including accounting for not applicable (NA) or unsure responses as nonresponses. An assessment was made of whether exclusion of particular survey data should be considered, based on issues found in areas including survey design, question type, or respondent data.

## 3 RESULTS

### 3.A Inclusion and exclusion of data and analysis

In the *standard* survey, data from Asia (n = 6) on reported DIR software (iii) were excluded due to insufficient data. Data on IR quality assurance mechanisms (vi) were excluded due to high rates of “none of the above,” with 35% for Americas (AMS), 50% for Asia 50%, 43% for Europe (EU), and 6% for ANZ (Table 10). In the extended survey, results on the number of datasets (xvi), the number of datasets used for commissioning by application (xvii), and patient‐specific QA (xviii) were excluded as there was no ability for respondents to skip questions that were not applicable to them and they were forced to input nominal values to questions.

### 3.B Respondent data

**Fig. 1:**
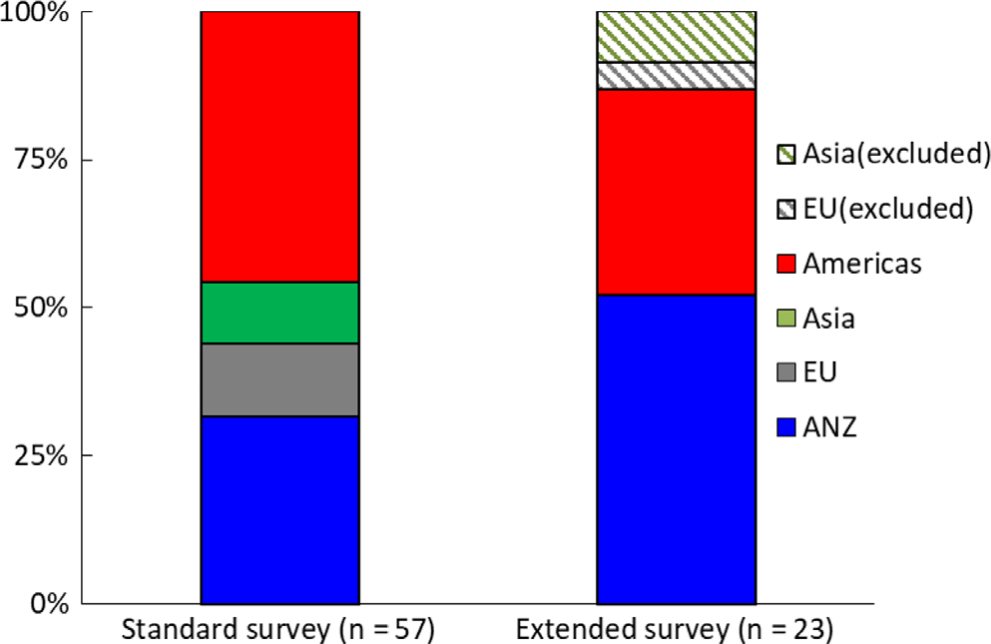
Standard survey (Left) with 26 responses from Americas, 18 from ANZ, 7 from EU, and 6 from Asia. Extended survey (Right) with 8 from Americas and 12 from ANZ; 1 response from EU and 2 from Asia were excluded from analysis (striped pattern). The color coding used (online version) involves ANZ blue, AMS red, Asia green, and Europe gray.

From the standard survey, 57 departments responded internationally [Fig. 1(a)] with data from each geographical region were analyzed. The extended survey had 23 international responses [Fig. 1(b)]: data from AMS (n = 8) and ANZ (n = 12) were analyzed but data from Asia (n = 2) and EU (n = 1) were not included in analysis at all due to very limited responses. Multi‐site and single‐site departments were registered as a single response. Fifty‐four percent of departments operated as a single site (AMS 38%, Asia 67%, EU 71%, ANZ 67%) and 60% were public sector departments (AMS 46%, Asia 67%, EU 86%, ANZ 67%). Departments had an average of five treatment units (range 1–24) over all treatment modalities (such as megavoltage and kilovoltage external beam therapy units, or brachytherapy machines).

#### 3.B.1 Department responsibilities

**Fig. 2:**
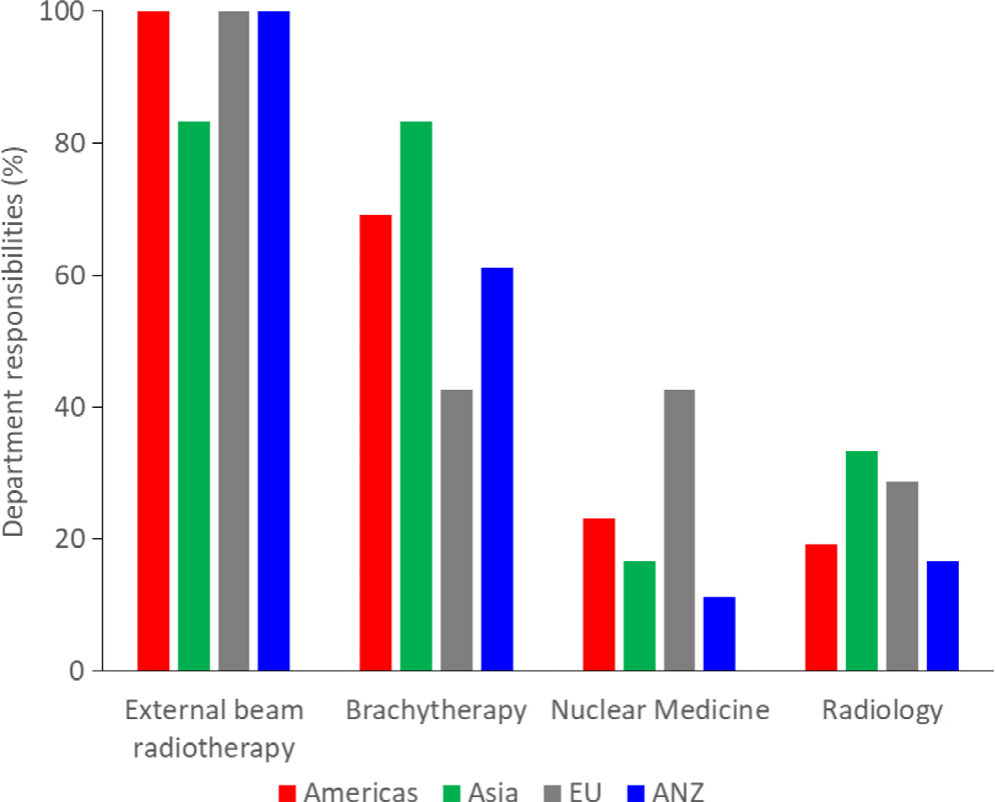
Department responsibilities for external beam radiotherapy, brachytherapy, nuclear medicine, and radiology by region.

Internationally, respondents noted their responsibilities with external beam radiotherapy (98%), 65% with brachytherapy, 21% with nuclear medicine, and 21% with radiology (see Fig. 2). Departments were also surveyed on university affiliation (international rate of 30%).

#### 3.B.2 Standalone DIR and RTPS DIR software

The combined international results on centers’ exposure to systems with DIR are presented in Table 1 (indirectly indicating clinical and/or research use). In 2018, dedicated DIR software was the most common (AMS 88%, EU 71%, ANZ 50%), followed by DIR‐enabled RTPS (AMS 12%, EU 43%, ANZ 33%), with some indications of multiple types of dedicated DIR software (AMS 12%, EU 29%, ANZ 28%). The least common pattern was a combination of dedicated DIR software with DIR‐enabled RTPS (AMS 4%, EU 14%, ANZ 11%). Use of DIR validation software was 12% internationally (AMS 8%, EU 29%, ANZ 11%) and 8% for open source DIR software (AMS 4%, EU 0%, ANZ 11%). The majority of departments reported exposure to some form of clinical DIR software (either dedicated DIR software or RTPS with DIR) in 2018 (AMS 92%, EU 71%, ANZ 61 %).

**Table 1:** International data of centers having exposure to systems with dedicated DIR software (SW), radiotherapy treatment planning system (RTPS) with DIR, and other DIR systems available in departments (%), with (o) denoting responses from open text.

Type of DIR SW	Software product	2013 INTL	2018 INTL	2023 INTL
Dedicated DIR SW	Velocity	14	41	45
	MIM	22	43	45
	Mirada	6	8	8
	Prosoma (o)		2	
	MRIdian (o)		2	
RTPS with DIR	Pinnacle	8	12	16
	Raystation	6	12	16
	Eclipse (o)		8	
	Brainlab (o)		4	
Open source DIR SW	Plastimatch (o)		6	
	Slicer (o)		4	
	ITK (o)		4	
	DIRART (o)		2	
DIR validation SW	ImSimQA	4	12	12
Any open source DIR SW	All SW		8	
Both dedicated DIR and RTPS with DIR	All SW	8	16	20
Multiple dedicated DIR SW	All SW	6	20	20
Any RTPS with DIR	All SW	12	24	29
Any dedicated DIR SW	All SW	33	73	75
Either dedicated DIR or RTPS with DIR	All SW	49	80	84

In 2018, data indicate that MIM^TM^ (MIM Software Inc., Cleveland, US) was prevalent internationally (43%), in AMS (54%), and ANZ (44%) but not in EU (0%). Velocity^TM^ (Varian Medical Systems Inc., CA, US) had international usage of 41% spread over AMS (46%), EU (43%), and ANZ (39%). Mirada^TM^ (Mirada Medical, Oxford, UK) had international usage of 8%, with most centers in EU (29%), some in ANZ (11%), and none in AMS. Internationally, 2% used Prosoma^TM^ (Oncology Systems Limited, Shrewsbury, UK) with 14% use in the EU. Among DIR‐enabled radiotherapy treatment planning systems (RTPS), Pinnacle3 Treatment Planning System with DIR (Philips, Amsterdam, Netherlands) had 12% INTL usage in 2018, with AMS (8%), EU (29%), and ANZ (11%). Raystation® with DIR (Raysearch Laboratories, Stockholm, Sweden) also had 12% of INTL use in 2018, with 12% in AMS, 17% in ANZ, but no uptake in EU. Eclipse^TM^ with DIR (Varian Medical Systems Inc., CA, US) had use of 7%, BRAINLAB^TM^ with DIR (Brainlab AG, Munich, Germany) 4%, and MRIdian*®* (ViewRay, Ohio, US) with DIR 2% internationally.

For dedicated DIR verification software, ImSimQA^TM^ (Oncology Systems Limited, Shrewsbury, UK) had 12% INTL in 2018, with 8% in AMS, 29% in EU, and 11% in ANZ. In 2018, there was a usage of open source DIR software of 8% internationally, with 4% in AMS, 17% in ANZ, and none in the EU. Among these, there was usage of Plastimatch^17^ of 5%, ITK^18^ 4%, Slicer^19^ 4%, and DIRART^20^ at 2%.

### 3.C Clinical adoption of rigid and deformable image registration

#### 3.C.1 Cumulative adoption of image registration techniques

**Fig. 3:**
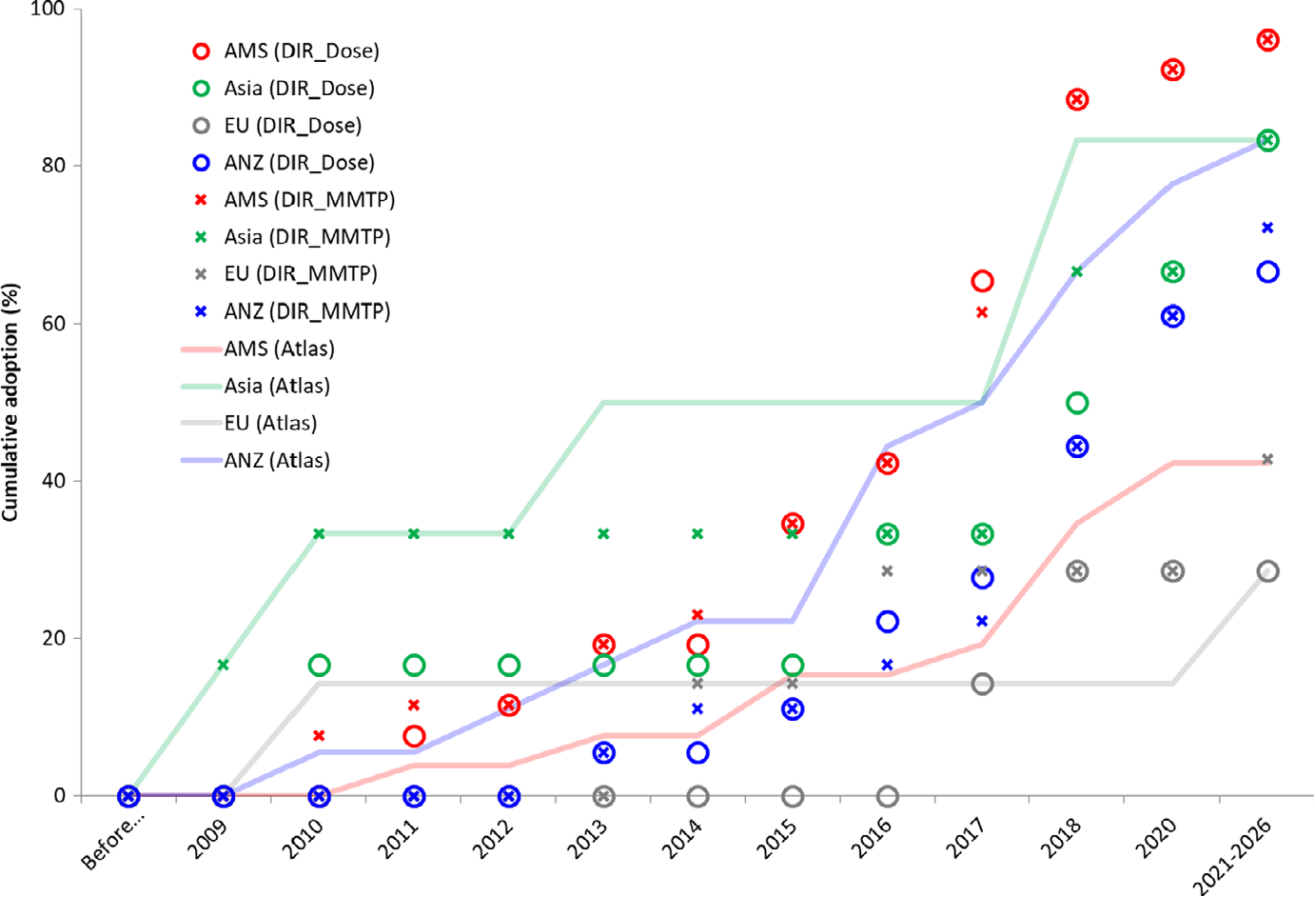
Cumulative adoption of atlas‐based segmentation (Atlas), deformable image registration with dose (DIR_Dose), and multi‐modality treatment planning (DIR_MMTP); note that data for 2018 and onward are indicative of respondent intentions and not actual adoption. Results are divided with color coding (online version) with ANZ blue, AMS red, Asia green, and Europe gray

Cumulative adoption curves indicate that the practice pattern for DIR is differentiated between use for atlas‐based segmentation, dose operations, and multi‐modality treatment planning (see Fig. 3). Adoption of DIR for dose (circle) and multi‐modality treatment planning (crosses) generally increased together, with a different pattern of atlas‐based segmentation (lines) uptake. Open text responses on adoption of DIR by IR technique are presented in Appendix S2.

#### 3.C.2 Uptake of RIR and DIR by image modality pair

Internationally, almost all respondents utilized CT‐CT with RIR (96%) with substantial use of DIR (51%). As RIR is an initialization step for DIR or could be used on its own, all RIR use values were higher than DIR. Ninety‐three percent used CT‐MR with RIR and 19% using DIR. Eighty‐one percent used RIR for CT‐PET with 47% using DIR. This data is nonspecific as to whether a planning CT is registered to the PET or the diagnostic CT. It is noted that a typical DIR process involves a DIR between the pCT and the dCT, with a further chain registration that utilizes a RIR of the dCT to the PET to display the pCT to the PET (similarly for SPECT). Eighty‐four percent used RIR for CT‐CBCT with 19% using DIR. Forty‐nine percent used RIR for MR‐MR with 5% using DIR. Nine percent used RIR for CT‐US with no respondent use of DIR for that combination (see Table 2).

**Table 2:** Utilization of rigid image registration (RIR) and deformable image registration (DIR) by image modality pair (%).

	Americas	Asia	EU	ANZ	INTL
CT‐CT					
RIR	92	100	100	100	96
DIR	77	33	43	22	51
CT‐MR					
RIR	92	83	86	100	93
DIR	27	33	29	0	19
CT‐PET					
RIR	73	83	71	94	81
DIR	77	50	0	22	47
CT‐CBCT					
RIR	85	83	100	78	84
DIR	15	33	43	11	19
CT‐US					
RIR	8	17	0	11	9
DIR	0	0	0	0	0
MR‐MR					
RIR	46	33	57	56	49
DIR	4	17	14	0	5

#### 3.C.3 Uptake of DIR by anatomical site

International data (AMS n = 8 and ANZ n = 12) on the uptake of DIR over anatomical sites are presented in Table 3. In AMS, DIR was most heavily used for head and neck, with 50% RIR vs 63% DIR for adaptive radiotherapy (ART), 100% RIR vs 88% DIR for multi‐modality treatment planning (MMTP), and 88% for both RIR and DIR when accounting for previous dose. Lung was the second site with most DIR use (50% ART, 63% MMTP, and 63% accounting for previous dose). Even with high DIR use in the head and neck and lung, the brain was a site where RIR dominated DIR use (13% vs 0% for ART, 88% vs 50% for MMTP, 75% vs 25% to account for previous dose). The pelvis, considered in this study to be a broad category for the pelvic region for both genders that also includes the prostate, represented a site where RIR dominated DIR use (13% vs 0% for ART, 100% vs 38% for MMTP, 88% vs 50% to account for previous dose).

**Table 3:** International data by anatomical site on the use of rigid (RIR) and deformable image registration (DIR) for multi‐modality treatment planning (MMTP), accounting for previous treatment (Prev Tx eval), adaptive radiotherapy (ART), and atlas‐based segmentation (%). Ratings of uncertainty of DIR with images and dose are in the last column (higher value represents more uncertainty, scaled from 0 to 100%).

	MMTP		Prev Tx eval		ART		Atlas‐based	Uncertainty
	RIR	DIR	RIR	DIR	RIR	DIR	segmentation	DIR
Brain	96	30	83	26	48	17	43	24
Head and neck	100	57	87	57	65	43	43	51
Breast	52	22	70	30	35	17	22	46
Lung	100	43	87	39	52	35	26	55
Esophagus	87	43	87	35	43	17	17	52
Pelvis	96	30	83	30	52	17	26	52
Prostate	96	30	87	35	48	22	30	48
Upper GI	83	30	83	30	39	17	17	56
Sarcoma	65	26	61	13	39	17	13	34
Hematological	39	13	48	13	26	9	9	25

The brain had the lowest levels of user‐reported ratings of uncertainty (AMS 26%, ANZ 22%). Other sites had increased levels of uncertainty: head and neck (AMS 63%, ANZ 42%), prostate (AMS 51%, ANZ 46%), breast (AMS 63%, ANZ 36%), esophagus (AMS 66%, ANZ 42%), pelvis (AMS 57%, ANZ 48%), and upper GI (AMS 74%, ANZ 44%). Nonresponse rates for uncertainty (respondents unsure) was on average 20% across anatomical sites, and was highest for hematological (AMS 63%, ANZ 25%) and sarcoma (AMS 50%, ANZ 25%).

#### 3.C.4 Staff involvement in rigid and deformable image registration

The extended survey captured respondents’ opinions on “ideal staff numbers for DIR” if the department had sufficient time and resources to train all staff (see Table 4). International practice varied with AMS featuring the highest levels of Physicist involvement, and ANZ the highest levels of RT involvement (AMS 30%, Asia 17%, EU 44%, ANZ 76%). Dosimetrists had higher levels of RIR involvement than Physicists in AMS (AMS 66%, Asia 17%, EU 33%) but lower in DIR than Physicists (AMS 51%, Asia 17%, EU 33%). RO involvement in RIR (AMS 33%, Asia 60%, EU 16%, ANZ 66%) was higher than DIR levels (AMS 28%, Asia 33%, EU 10%, ANZ 19%) with a large difference relative to ideal DIR involvement (AMS 55%, ANZ 77%). A gap between existing and ideal levels of DIR involvement existed for all staff groups, with gaps in ANZ larger than AMS for Physicists (ANZ gap 53% vs. AMS gap 16%) and RTs (ANZ gap 62% vs. AMS gap 10%).

**Table 4:** Staff involvement across geographical continents for rigid image registration (RIR) and deformable image registration (DIR) at current levels based on standard survey (n = 57); data on ideal levels were based on extended survey (n = 23) which had limited data from Asia and EU. Note that NA indicates lack of extended survey data of ideal levels of DIR staff involvement. Note that Dosimetrist is not applicable for ANZ, and is omitted from the table.

	RIR current	DIR current	DIR ideal
Radiation Oncologist			
ANZ	66	19	77
AMS	33	28	55
ASIA	60	33	NA
EU	16	10	NA
Medical Physicist			
ANZ	38	23	77
AMS	56	57	73
ASIA	50	27	NA
EU	47	39	NA
Radiation Therapist			
ANZ	76	21	83
AMS	30	12	23
ASIA	17	13	NA
EU	44	13	NA
Dosimetrist			
AMS	66	51	80
ASIA	30	17	NA
EU	44	33	NA

### 3.D Implementation and operational characteristics

#### 3.D.1 Image registration training

Training data are presented in Table 5. Internationally, formal training regimens were uncommon for IR (INTL 7% for RO, 11% for RTs, 12% for RO, and no data on Dosimetrists). Anatomical site‐specific training was low (INTL 12%). Self‐training with standard operating procedures (INTL 33%), online materials (INTL 33%), and vendor material (47%) were used in conjunction with informal peer training (INTL 70%) and self‐assessment of competency (65%). Vendor‐based training was common (INTL 67%).

**Table 5:** Data from image registration training question.

Respondent responses (%) per continent	Americas	Asia	EU	ANZ
Self‐training with vendor material	65	33	29	33
Self‐training with online material	46	33	0	28
Self‐training with standard operating procedures	27	33	29	44
Informal peer training	58	33	71	100
Vendor training	85	33	29	67
Competency based assessment — self assessed	73	50	57	61
Competency based assessment — trainer assessed	0	0	14	33
Competency based assessment — written exam	0	0	0	0
Competency based assessment — practical exam	0	0	0	0
Clinical training guide for trainees	0	0	0	6
Training program for RO	0	33	0	11
Training program for RT	0	0	14	33
Training program for Physicist	8	17	14	11
Anatomical site specific training	0	17	14	28
No formal training program	15	33	0	0

#### 3.D.2 Image registration processes evaluation

**Fig. 4:**
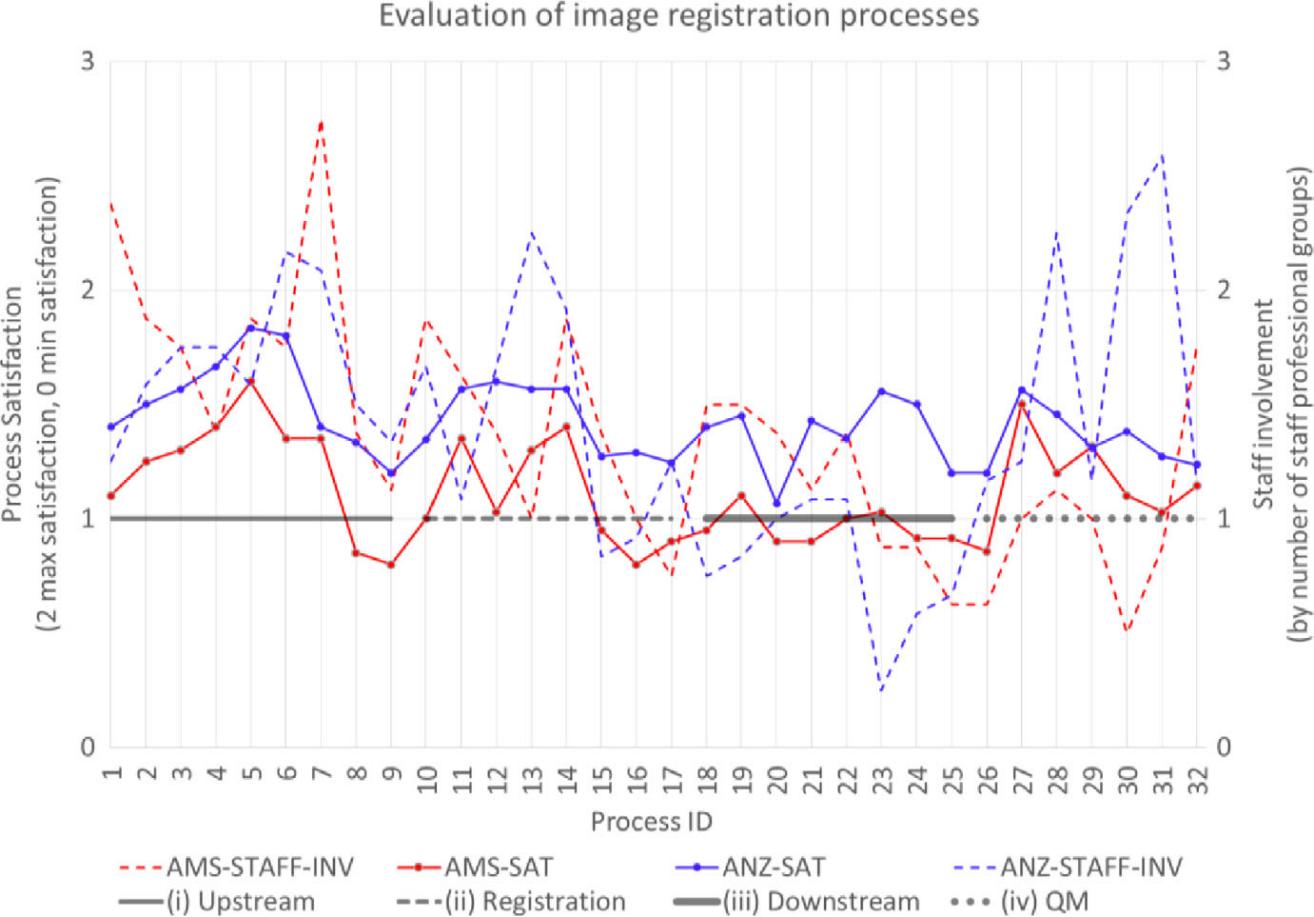
Evaluation of image registration process by relative measures of satisfaction (below 1 deemed unsatisfactory — left side) and staff involvement (below 1 on average having some processes without staff involvement — right side); processes grouped by (i) upstream (1–9), (ii) registration (10–17), (iii) downstream (18–25), and (iv) management (26–32).

The extended survey captured process performance by relative satisfaction and number of staff group involvement (see Fig. 4). Average number of staff group involvement for upstream processes (AMS 1.8, ANZ 1.7) and registration processes (AMS 1.4, ANZ 1.4) were higher than downstream (AMS 1.2, ANZ 0.8). Averaged relative satisfaction of processes for upstream processes (AMS 1.2, ANZ 1.5) and registration processes (AMS 1.1, ANZ 1.4) were similarly higher than downstream processes (AMS 1.0, ANZ 1.4). For management processes, relative average satisfaction was similar in AMS and ANZ (AMS 1.2, ANZ 1.3) while staff group involvement varied (AMS 1.0, ANZ 1.7). The lowest level of staff group involvement in AMS was in‐house software engineering (0.5); in ANZ, it was for checking of atlas‐based segmentation (0.3) and the process to validate deformed image and dose (0.6). The lowest level of relative satisfaction in AMS was for explicit registration prescription and documentation of uncertainties (0.8 each); for ANZ, the lowest satisfaction was with processes in decision when registrations had risk of deformation (TG132 registration accuracy^4^ level 2), with a score of 1.1. Average rates of N/A responses for registration (AMS 2%, ANZ 15%) and downstream (AMS 5%, ANZ 41%) were higher than for upstream (1% each) or management (AMS 14%, ANZ 8%). Processes with highest N/A in AMS for in‐house software engineering (50%) and for ANZ were with the process to validate deformed doses and images (67%).

#### 3.D.3 Image registration challenges

The standard survey captured key challenges of respondents by continent (see Table 6). Internationally, 38% respondents reported challenges in upstream processes, 44% for registration challenges, 39% for downstream challenges, and 16% for management challenges. For upstream processes, reported challenges were 47% for *image quality*, 39% for *cropped images*, 37% for communications on intended use and registration technique, 30% on image selection, and 28% on registration landmark required. For registration processes, reported challenges were 47% on *determining when registration was satisfactory*, 46% on *quantitative QA for DIR*, and 39% on qualitative QA for DIR. For downstream processes, reported challenges were 44% on *actions for unsatisfactory registrations*; with 33% for documentation and/or appropriate follow up of registrations. For management processes, reported challenges were 26% for *image transfer across multiple systems*, 18% for image infrastructure (archive, backup, etc.), 18% for image/software accessibility, 14% for definition of roles, and 7% for in‐house software engineering.

**Table 6:** Survey data on key challenges for rigid and deformable image registration (DIR).

Respondent responses (%) per continent	Americas	Asia	EU	ANZ
Image quality issues (resolution, contrast, etc.)	42	50	71	44
Determining when registration satisfactory	50	50	43	44
DIR Quantitative QA of ensuring deformation is OK	58	17	43	39
Determining action when registration unsatisfactory	50	50	14	44
Image cropped (scan length, field of view, etc.)	35	33	57	39
DIR Qualitative QA of ensuring deformation is OK	46	17	43	33
Communication on intended use and technique	35	33	43	39
Documentation of registration accuracy and follow‐up	35	33	14	39
Selecting the appropriate image	31	17	14	39
Insufficient training, trained staff availability	23	33	14	44
Determining which registration landmark required	35	33	14	22
Image transfer (import/export) of multiple systems	15	33	29	39
Image infrastructure (storage, backup, etc.)	12	0	14	33
Image or software accessibility	12	0	29	28
Insufficient definition of roles	12	33	14	11
In‐house software engineering	4	0	0	17

#### 3.D.4 Evaluation of image registration methods

**Fig. 5:**
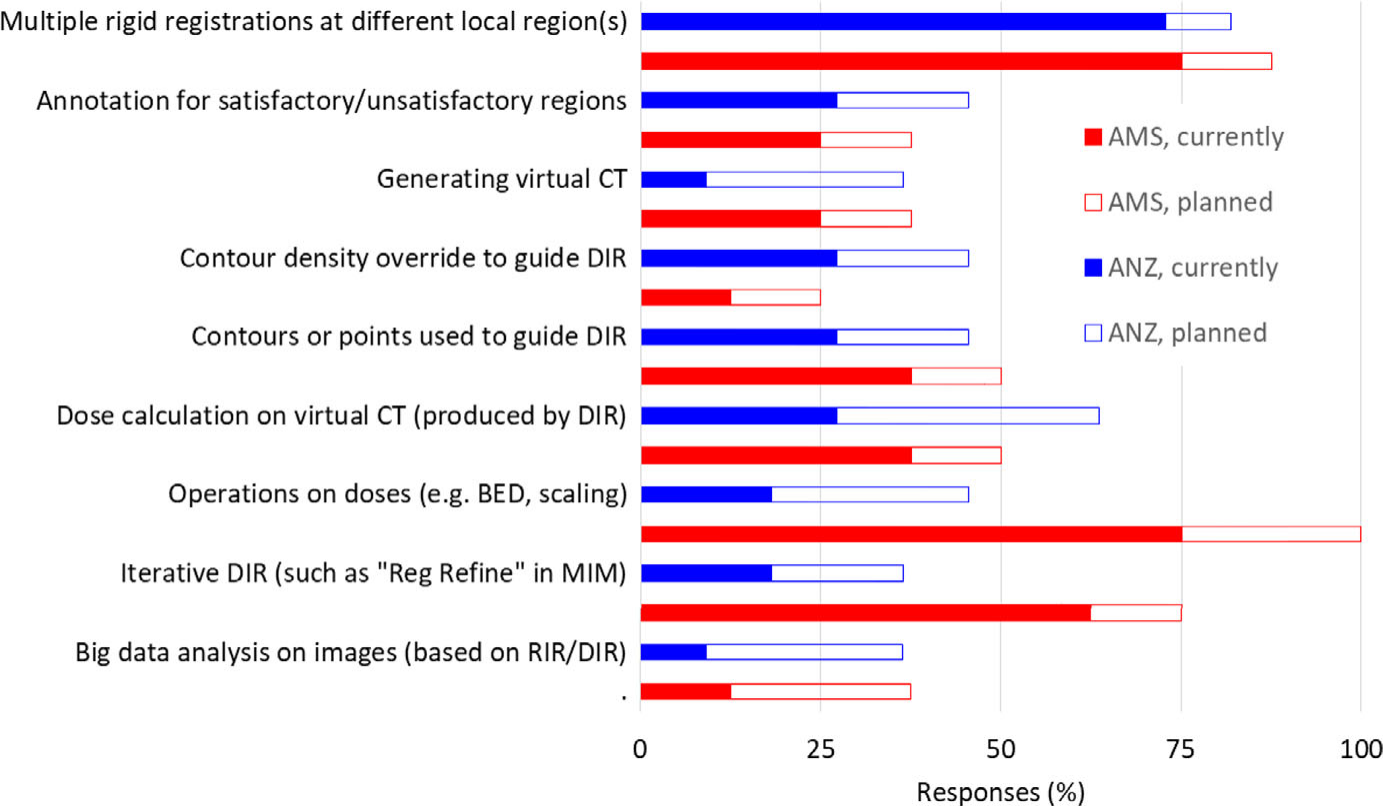
Evaluation of variation in nonstandard image registration operations, both currently used (2018) and planned for the future; BED refers to biologically effective dose.

The extended survey captured how nonstandard IRs are performed, and how they would be performed based on respondent plans (see Fig. 5). Internationally, there was (as of 2018) 74% respondents performing multiple RIR at different local region(s), with plans for this to reach 84% in the future. In terms of more advanced DIR, iterative DIR techniques were the most common (current 36%, future 52%), followed by use of contours/points to guide DIR (current 31%, future 47%), as well as contour masking to guide DIR (current 21%, future 37%). In terms of related DIR functions, dose propagation with DIR (current 31%, future 58%) was more common than using DIR for HU overrides such as generation of synthetic CT from CBCT (currently 15%, future 37%). Ancillary tasks including dose operations, such of dose to account for incomplete delivery of radiotherapy fractions was relatively common (current 41%, future 67%). In terms of quality assurance, annotations to document regions of registration accuracy (or lack of) were uncommon (current 26%, future 42%). Big data projects reliant on registrations (RIR or DIR) were uncommon (current 10%, future 37%).

#### 3.D.5 Evaluation of image registration quality assurance metrics

The extended survey captured IR assessment methods and metrics, stratified by staff and by continent (See Table 7). This indicates that qualitative assessment is more common than quantitative assessments for each region (AMS vs ANZ) and for all staff groups (RO, RT, Physicist, Dosimetrists). All staff groups utilized qualitative assessment; the most common forms being visual evaluation (INTL 66%, AMS 56%, ANZ 72%), comparison with contours (INTL 51%, AMS 44%, ANZ 56%), and subjective considerations (NTL 55%, AMS 53%, ANZ 56%); less common were use of rulers/grids (INTL 35%, AMS 25%, ANZ 42%) or screenshots (INTL 23%, AMS 13%, ANZ 31%). There were staff and regional variation in the use of quantitative methods, primarily by physicists (AMS 15%, ANZ 22%) but also with dosimetrists (AMS 13%), therapists (AMS 0%, ANZ 10%), and RO (AMS 0%, ANZ 7%). The most common quantitative metrics were target registration error and mean distance to agreement (INTL 14% each), followed by dice similarity coefficient (INTL 11%), Jacobian (INTL 8%), and consistency (INTL 7%).

**Table 7:** Data on image registration quality assurance methods and metrics (QL denotes qualitative, QT denotes quantitative).

	% divide per region	Americas				ANZ		
		RO	Phys	RT	Dos	RO	Phys	RT
QL	Anatomical landmarks visualized	50	63	38	75	67	67	83
	Anatomical landmarks with screenshots	13	25	0	13	17	42	33
	Anatomical landmarks with grid/ruler	25	38	13	25	25	50	50
	Comparison with contours	50	50	25	50	33	67	67
	Subjective considerations	63	75	25	50	75	33	58
QT	Target registration error (TRE)	13	13	0	13	17	17	17
	Mean distance to agreement (MDA)	0	13	0	13	8	33	17
	Dice similarity coefficient (DSC)	0	13	0	13	0	33	8
	Jacobian	0	13	0	13	8	8	8
	Consistency (transitivity)	0	25	0	13	0	17	0

#### 3.D.6 Image registration validation datasets

Internationally (AMS and ANZ), the extended survey captured data on the types of validation datasets used (see Table 8), by image modality and by subcategory of dataset types (clinical, physical, and digital). The most common validation datasets by image modality were for CT (38% averaged over all dataset types), CBCT (28%), MR (23%), PET (18%), 4DCT (18%), 4DCBCT (16%), and the least common was US (9%). For digital datasets, there were similar rates of deformable (15% averaged over all image modalities) and rigid (14%); while for physical datasets, rigid datasets (34%) were more common than deformable physical phantoms (6%), which had the lowest value among all dataset types. For clinical datasets, retrospective (39%) was more common than prospective (21%) data (Tables 9, 10, 11).

**Table 8:** International data (AMS and ANZ extended survey) with percentage (%) of respondents having datasets of a particular category (by image modality, and subcategories from digital, physical, or clinical dataset types); *note that validation clinical datasets did not specify whether it was directed toward RIR or DIR validation.

	Digital datasets		Physical datasets		Clinical datasets* (Rigid and/or Deformable)	
	Rigid	Deformable	Rigid	Deformable	Retrospective	Prospective
CT	30	40	60	15	60	25
MR	20	15	35	5	40	20
PET	20	15	20	0	35	20
CBCT	20	15	45	5	55	30
US	5	5	15	0	20	10
4DCT	5	10	30	10	35	20
4DCBCT	0	5	30	10	30	20

**Table 9:** A list of abbreviated image registration processes used in this study.

Process category	Process ID	Process description
Upstream	1	Processes to ensure sufficient information in image
	2	Processes to ensure Setup factors between images optimized
	3	Processes to ensure patient factors between scans optimized
	4	Processes to ensure image quality optimized
	5	Processes for satisfactory orientation and data integrity
	6	Processes to ensure correct image registered
	7	Processes to validate incorporation of previous RT information
	8	Processes for Implicit registration prescription (e.g., protocol defining what landmark to register to)
	9	Processes to prepare explicit registration prescription (e.g., RO writing patient specific instructions on what landmark to register to)
Registration	10	Processes for interpreting implicit/explicit registration prescription
	11	Registration technique optimal (rigid)
	12	Registration technique optimal (deformable)
	13	Processes to ensure image quality optimized (e.g., artifacts)
	14	Landmarks in image identified
	15	Registration QA
	16	Uncertainty/issues documented
	17	Accuracy level documented and reported
Downstream	18	Registration results interpreted
	19	Decision when whole scan or local regions aligned
	20	Decision when usable with risk of deformation (additional PTV margin may be required as per TG 132)
	21	Processes to calculate and apply margin policy
	22	Process followed when image registration is usable for diagnosis only or not for clinical use
	23	Process to ensure atlas‐based contours checked/edited/finalized
	24	Process to process/validate deformed image and dose
	25	Process to document registration QA and actions in hospital database
Management	26	Managing roles/responsibilities with allocating time from trained staff to known task times
	27	Quality management of imaging equipment
	28	Sufficient datasets, validation, and procedures in place
	29	Coordination and integration with RO, RT, Physics, as well as various portfolios. Also with Nuclear Medicine, Radiology, Medical Oncology, etc.
	30	In‐house software engineering or use of advanced vendor functions
	31	Project management: balancing quality, risk, and efficiency
	32	Reactive systems: all technical and human issues, incidents and near misses are managed

**Table 10:** Survey data on image registration quality assurance mechanism.

Respondent responses (%) per continent	Americas	Asia	EU	ANZ
Formal QA check task in system	19	17	14	56
Registration instruction in protocol	15	33	14	61
Registration instruction prescribed by RO	19	33	14	28
[DIR] Qualitative or quantitative check of DVF and deformed image	35	0	43	17
Registration QA form with achieved accuracy level documented	15	0	14	28
Decision tree or equivalent	8	0	0	11
None of the above	35	50	43	6

**Table 11:** Summary of DIR software available by software type and whether the survey captured the data directly (yes), indirectly (open response), or not at all (no).

DIR software type	DIR software	Website	Survey data
Commercial dedicated DIR SW	ProSoma	http://www.medcom‐online.de/clinical‐areas‐products/teletherapy/prosoma/	Yes
	Velocity	https://www.varian.com/oncology/products/software/velocity	Yes
	MIM	https://www.mimsoftware.com/	Yes
	Mirada	https://mirada‐medical.com/	Yes
Commercial DIR validation SW	ImSimQA	https://www.osl.uk.com/ImSim	Yes
Commercial RTPS with DIR	Brainlab Elements	https://www.brainlab.com/surgery‐products/overview‐neurosurgery‐products/brainlab‐elements/	Open response
	Eclipse	https://www.varian.com/oncology/products/software/treatment‐planning/eclipse‐treatment‐planning‐system	Open response
	MRIdian	https://viewray.com/discover‐mridian/	Open response
	Pinnacle	https://www.usa.philips.com/healthcare/solutions/radiation‐oncology/radiation‐treatment‐planning	Yes
	RayStation	https://www.raysearchlabs.com/raystation/	Yes
	iPlan	https://www.brainlab.com/radiosurgery‐products/iplan‐rt‐treatment‐planning‐software/	No
	Monaco	https://www.elekta.com/software‐solutions/treatment‐management/external‐beam‐planning/monaco.html	No
	iVAS	http://www.item‐corp.jp/en/	No
	Accuracy Precision	https://www.accuray.com/software/precision‐treatment‐planning/	No
	DOSIsoft	https://www.dosisoft.com/	No
Open source DIR SW	Plastimatch	https://www.plastimatch.org/	Yes
	Slicer	https://www.slicer.org/	Yes
	ITK	https://itk.org/	Yes
	DIRART	https://code.google.com/archive/p/dirart/	Yes
	MEVISLab	https://www.mevislab.de/	No
	Advanced normalization tools (ANT)	http://stnava.github.io/ANTs/	No
	NiftyReg	https://sourceforge.net/projects/niftyreg/	No
	Elastix	http://elastix.isi.uu.nl/	No
	DRAMMS	https://www.nitrc.org/projects/dramms/	No

#### 3.D.7 Image registration request and report form

**Fig. 6:**
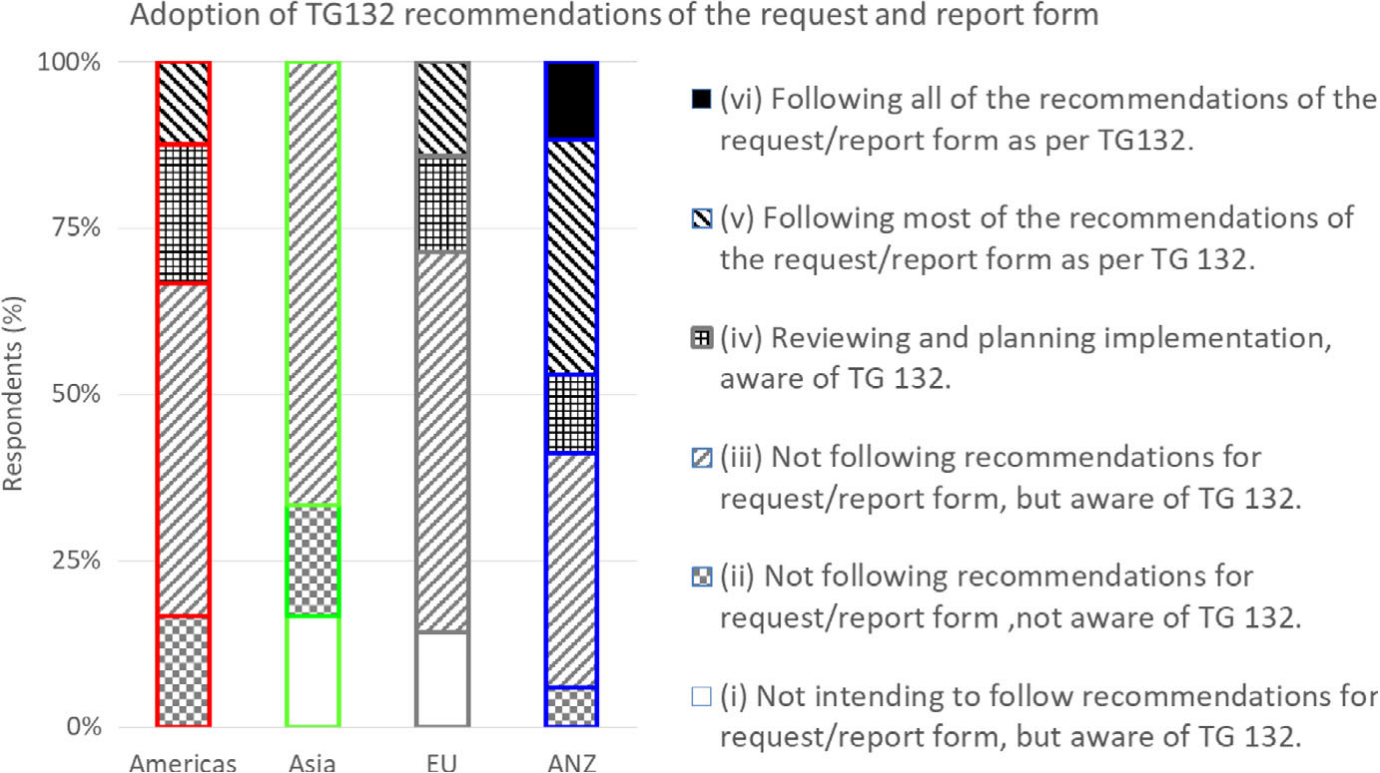
Evaluation of adoption of AAPM Task Group 132 (TG132) recommendations of the request and report form for each continent, from (i) not intending to follow recommendations for the request/report form to (vi) all recommendations followed for the request/report form.

Figure 6 shows the level of awareness of the TG132 report with the specific adoption of the IR request and report form. As of the survey start (April 19, 2018) and the TG 132 report publication (May 23, 2017), international awareness of the report was common (INTL 82%) in all continents (AMS 83%, Asia 67%, EU 86%, ANZ 94%). However, adoption of most recommendations for the IR request and report form was not common (INTL 18%), with only Australasia having a relatively high adoption rate (AMS 13%, Asia 0%, EU 14%, ANZ 47%).

### 3.E Quality, safety, and value in the implementation of image registration

#### 3.E.1 Criteria for commissioning and implementing image registration

Respondents were queried about what considerations they took into account in the commissioning and implementation of DIR, and chose the three most important criteria from a list.

Survey results (n = 20, AMS = 8, ANZ = 12) indicated that the most commonly nominated criterion was a quality system to enable measurement and improvement (overall 55%, AMS 75%, ANZ 42%). The next most commonly nominated considerations were: effective optimization of registration quality and QA (overall 45%, AMS 25%, ANZ 58%); rapid efficient progress and clinical release (overall 45%, AMS 25%, ANZ 58%); documentation and management of uncertainties/risks (overall 45%, AMS 50%, ANZ 42); feasibility for tasks to be practical and achievable (35%); roles and training to be specified and managed (25%); ongoing and recurrent human and financial costs (20%); compliance and best practice (10%); documentation of registration quality and error handling (10%); and proactive system validation and risk management (5%).

#### 3.E.2 Measures of quality and safety for image registration

Open responses on the measures of quality for RIR were: (i) risk/incident entries, (ii) physical, (iii) digital or phantom measurements, (iv) patient‐specific QA, (v) treatment outcomes based on toxicity, (vi) qualitative IR checks, (vii) quantitative IR checks, (viii) peer review, and (ix) user feedback. The responses for DIR added details including (x) specific QA check on DIR by physics and RO, (xi) department quality improvement system, (xii) that it depended on application, (xiii) that application of best practice such as TG132 report, (xix) staff confidence, (xx) audit results, and (xxi) specific pathways for tracking DIR. Open responses on measures for ensuring safety of RIR and DIR (e.g., how would incidents be detected) had similar responses, but also included (xxii) audits and (xxiii) offline image reviews, and (xxiv) having specifically trained staff QA DIR. General comments on the quality and safety of DIR noted the (a) large learning curve for DIR, (b) that DIR is high risk due as it is difficult to evaluate even for experts as well (c) having dangerous consequences, (d) that a conservative and cautious approach is required for deforming dose, (e) that there are limits in current DIR algorithms, and (f) that suitable controls with stop/go charts need to be implemented in clinical practice to minimize risk.

#### 3.E.3 Evaluation of value in implementation of DIR with risk‐benefit rating by use case

**Fig. 7:**
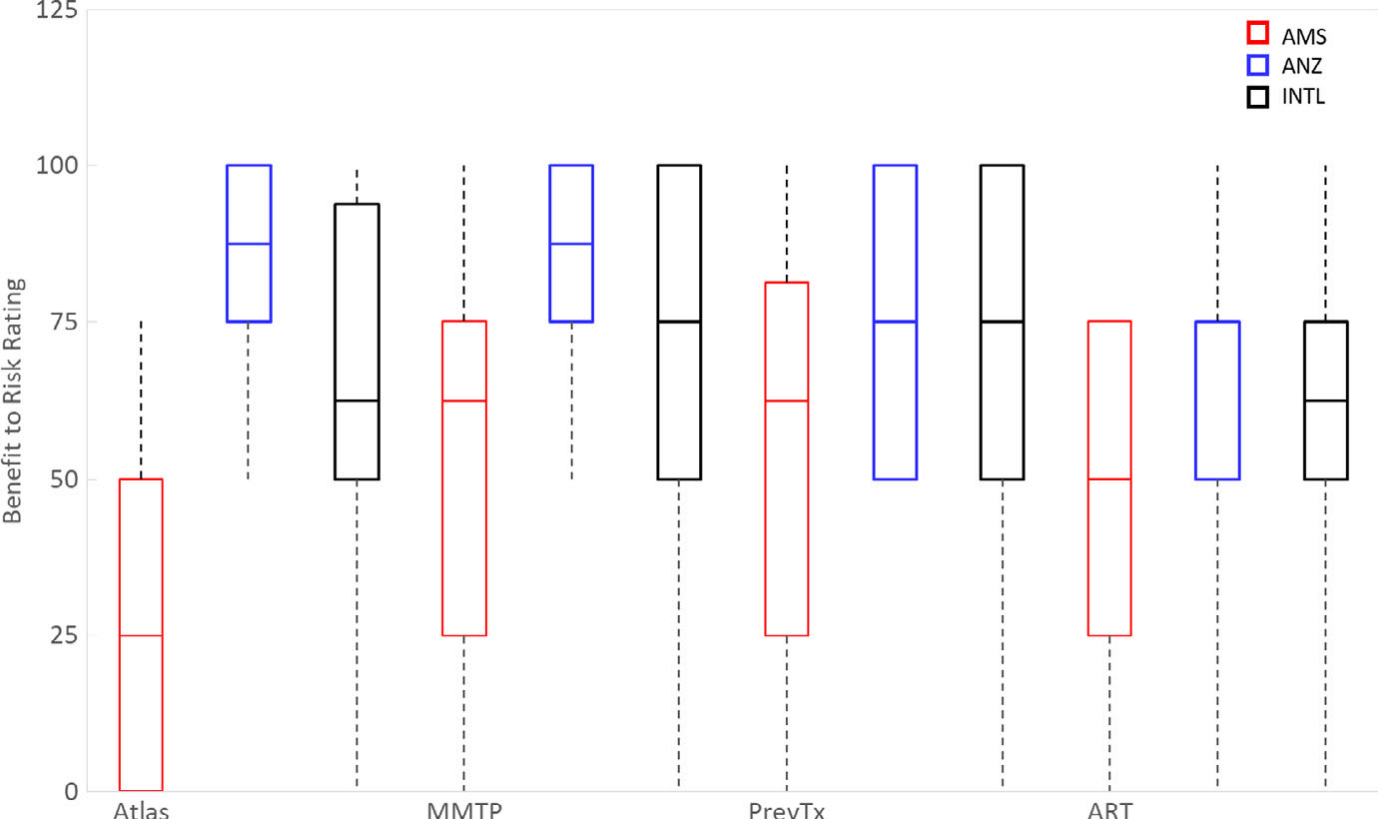
Box plot of benefit to risk rating of Americas (AMS, red). Australasian (ANZ, blue), and International (INTL, black) perceptions of the value of atlas‐based segmentation, use of DIR for multi‐modality treatment planning (MMTP), use of DIR for accounting for previous treatment (PrevTx), and adaptive radiotherapy (ART); Benefit to risk rating of 100% indicates that the benefits outweigh the risks significantly.

The International data (n = 20, AMS = 8, ANZ = 20) shown in Fig. 7 indicate that based on averaged values, the DIR functionality with most value were for multi‐modality treatment planning (INTL 69%, AMS 53%, ANZ 83%); followed by accounting for previous treatment and response assessment (INTL 67%, AMS 56%, ANZ 75%). The value of DIR for adaptive radiotherapy (defined as any use of DIR for ART, without specifying processes) was nominated at 57% (AMS 47%, ANZ 66%) which is close to parity (risks balanced against benefits). Atlas‐based segmentation had significant variance in value ratings (INTL 58%, AMS 28%, ANZ 83%).

## 4 DISCUSSION

This practice pattern data provides useful insights into the international status of rigid and deformable image registration implementation from 2008 to 2018. Historical evidence of the first uses of all DIR is presented along with the current and planned future adoption rates (Fig. 3). Batumalai et al. sampled 33 out of 46 ANZ centers to obtain a rate of 22% for DIR‐based multi‐modality treatment planning in August–October of 2015,^14^ compared with our data (18 ANZ centers) of 11% (2015) to 17% (2016). Kisling et al. reported an international rate of 26% for DIR with composite doses in 2016,^16^ which matched our rate of international value from our International data (INTL 26%, AMS 42%, Asia 33%, EU 0%, ANZ 11%). In 2018, Kadoya et al.^15^ surveyed Japanese practice and characterized the use of deformed images (56%) and dose accumulation (73%) which was within 10% of International averages from this study (65% and 63% on DIR for MMTP and for deformed dose, respectively); similarly, the Japanese use of DIR for segmentation (63%) and propagation (53%) are in broad agreement with our international data of 47% for atlas‐based segmentation. In the United States, adoption of IGRT started around 1999 and 10 yr passed until it became mainstream in 2009^13^; our data show international adoption of DIR also started around 2009 with significant growth in a decade, in parallel with the adoption of IGRT. The survey data suggest that most respondents intended to implement DIR for dose and multi‐modality treatment planning by 2020–2026.

Even when DIR is an option, RIR use is dominant for the brain and pelvis, which could be considered sites where the overall advantages of RIR outweigh DIR. For example, while RIR may be considered sufficient for brain^21^ due to the relatively nondeformable nature of the skull, DIR for the prostate involves multiple complexities such as low contrast in soft‐tissues,^21^, ^22^ bowel gas,^23^ potential biomechanical models,^5^ sliding tissues,^6^ and multiple treatment modalities (e.g., brachytherapy). This correlated with the 2018 Japanese DIR survey which named prostate as the site where DIR performs the worst.^15^ For the brain, RIR could be used in multiple ways such as for MMTP, to account for previous dose, and ART. ART can be simple or complex with either RIR or DIR,^24^ and recent survey data of ART (using either RIR or DIR) in India correlates with this survey showing relative preference for use in the head and neck (92%) and lung (52%).^25^ The data indicate that the characteristics of image differences across anatomical site can drive DIR use: for example, lower levels of uncertainty in the brain indicated that RIR was considered most appropriate; head and neck had the second lowest score of uncertainty which was among anatomical sites where DIR was most prevalent in our data. Clinical uptake may correlate to DIR performance as published by multi‐institutional studies such as for head and neck,^26^, ^27^ lung,^28^ thorax,^27^, ^29^ and pelvis.^27^ However, survey results of DIR use over anatomical region indicate that DIR algorithms^4^ were considered inherently not robust enough for general validation^30^ and use.^8^ In scenarios where DIR is challenging (e.g., tissue appearance or disappearance^6^, ^31^ or large deformations^6^), accurate use requires appropriate management of uncertainties.^4^

Data on the use of RIR with *multi‐modality* imaging show the relative dominance of CT‐based imaging with different CT scans (planning CT to diagnostic CT, or to CBCT), with decreasing use of MR (CT‐MR or MR‐MR) and US (CT‐US or US‐US). The imaging data used, particularly primary planning images for plan generation but also diagnostic and treatment imaging, could be a major factor in how centers utilized DIR. This survey data agrees with the Japanese data in 2018^15^ which found single modality DIR (CT‐CT) as the most common DIR image modality pair. Lower DIR usage of CT‐CBCT usage could correspond to additional complexities such as image noise and streaking^32^, ^33^ for contour propagation, or image truncation^32^, ^34^ for synthetic CT generation^35^ which may creep into the implementation of adaptive radiotherapy.^24^, ^36^, ^37^

Data indicate that more centers have independent DIR software than RTPS‐based DIR software. Department requirements for DIR software may depend on factors including imaging modalities, treatment modalities, and required applications. In addition to financial costs of software licenses, there may be time and resources required for ensuring specialized staff and validation equipment can support the safe operations and quality assurance of such functions. While the exact numbers of DIR software types and licenses was not surveyed, minimum and optimal levels of software would depend on the department specific merits (and costs) of (a) independent DIR software flexible for all image data, (b) treatment focused DIR RTPS software, or (c) research or validation DIR software. As departments plan the scope of DIR implementation (e.g., accuracy and uncertainty balance of RIR vs DIR), strategic planning considerations of the product life cycles of each available solution^38^ could be important.

This study provides data on awareness of the TG132 report (INTL 82%) as well as insight into the lack of widespread adoption of the IR request and report form (INTL 18%), which may highlight a community preference against the generation, communication, and management of formal documentation for IR patient‐specific QA. Three different approaches of evaluating IR processes (process satisfaction, staff involvement, key challenges) found that key concerns were registration accuracy classification (satisfactory or not) with appropriate follow‐up use. Staff factors for IR potentially increases with iterative RIR and DIR (Fig. 5); the dominance of qualitative over quantitative QA (Table 7); and substantial variations in staff roles across institutions (Table 4). Formal multidisciplinary IR training programs (currently lacking, as per Table 5) based on case studies with qualitative and quantitative metrics could improve the general performance of departmental IR techniques.

While Physicists may not necessarily be routinely involved in RIR, survey data suggest that they have the highest involvement in DIR of all professions, which is in agreement with the findings of a recent DIR survey in Japan.^15^ While Physicists may be only indirectly involved in operations they are directly responsible for assessing the performance the IR algorithms and techniques, and provide clinical guidance on associated actions and tolerances. Radiation Oncologists are expected to have a key role in identifying clinical aims of IR, specifying the registration accuracy required, and ensuring the safe and appropriate use of registration.^4^, ^39^ RTs and Dosimetrists are strongly involved in imaging before IR, have a key role in RIR, and a growing role in DIR. Survey data indicates that multidisciplinary peer review and audits would facilitate the safe operations of DIR, particularly when trained staff have responsibilities for managing DIR accuracy^4^, ^5^, ^30^ supported by departmental safety, risk, and incident reporting processes.

Data on implementation criteria show that the priority is for a quality system based on registration accuracy, which requires trade‐offs in documentation or management of uncertainties (AMS priority) with a reduction in efficiency and rapid progress (ANZ priority). Due to the inherent limitations of both RIR and DIR, management of IR uncertainties may be required as routine practice,^4^ employing practical solutions such as automated generation of documentation. Data on benefit to risk ratings, which can determine whether a technique should be used in a department, showed that the largest benefit/risk ratio was for accounting for previous dose (most worth implementing), followed by MMTP, adaptive radiotherapy, and then atlas‐based segmentation. This could be due to the increased complexity of group‐wise registration with adaptive radiotherapy (up to a registration per treatment image)^24^ compared to re‐irradiations, where registration could be pair‐wise.^8^ Variability in benefit to risk ratings reflects conflicting positions of minimizing risks (safety) vs implementing DIR to increase accuracy (benefits).^40^ This could be addressed with practical risk‐based solutions that systematically address risks^9^ or increase benefits, with clinical translation appropriate when benefits outweigh risks.^41^

The survey captured the use of most common commercial DIR software^15^; however, the open text responses listed eight additional DIR software products that were not in the survey question. A more complete list of all software found in the literature is provided (Table 11). Other limitations of the survey were limited time for survey data collection as it was required for an associated workshop, limited international representation, and practical limits on participant time spent filling in the survey. At the time of survey design, the authors did not include deep learning (DL) applications as an alternative to DIR algorithms; while there were more publications of DL techniques than DIR in 2018,^42^ there was a lack of commercial DL software. Commercial DL contours have demonstrated superiority over atlas‐based segmentation in accuracy and efficiency^43^; while there are increasing and promising trends in DL‐based IR, challenges remain^44^ and commercially DL IR are currently unavailable.

There are various limitations to the survey, including limited responses due to the large number of questions (29 standard and 81 extended). This reduces accuracy and introduces potential systematic bias^45^ such as the potential underestimation of DIR adoption in EU (7 standard and 1 extended response) due to the low response rate or overestimation with centers not practicing DIR choosing not to participate in the survey. The categorization of data by continent limits the usefulness of data for a particular region. Despite various limitations such as in question design or responses, the survey results offer insights and data not available elsewhere.

Future work could include a focused survey on multidisciplinary use of DIR carried out in cooperation with national or organizational stakeholders. This could be linked to multi‐institutional scientific study of DIR performance based on shared datasets where DIR corrections range from being beneficial to contraindicated.^31^, ^46^ While promising research such as deformable physical phantoms^47^, ^48^, ^49^, ^50^ and automated deformable vector field analysis^51^, ^52^ offer technical solutions, practical validation of registration^4^ may require a combination of qualitative with quantitative approaches.^4^, ^6^ Similarly, it may be prudent to focus in parallel on optimizing the safe use of commercially available solutions for achievable improvements now, as well as development for increasingly complex registration techniques, such as predictive models of dose accumulation, online replanning, and functional guidance.^24^

## 5 CONCLUSION

An international survey was performed of radiotherapy departments in the Americas, Asia, Europe, and Australia/New Zealand. Practice pattern data on DIR software and RIR/DIR utilization for a range of use cases and anatomical sites were obtained. This provides valuable insight into implementation patterns of IR, enabling the development of a coherent strategy for clinical adoption. Practice pattern data from this survey could be used to develop institutional or regional best practice guidelines for the safe and effective use of IR in radiotherapy.

## CONFLICT OF INTEREST

No conflict of interest.

## Supporting information


**Appendix S1.** Practice pattern survey questions (2018 Google Forms), clarification, and limitations.

**Appendix S2.** Open responses to adoption of DIR by use case.
