# Supplementary material for: An international survey on the clinical use of rigid and deformable image registration in radiotherapy
Source: J Appl Clin Med Phys. 2020 Sep 11;21(10):10–24. doi: 10.1002/acm2.12957 (PMC7075391; doi:10.1002/acm2.12957)
Supplement: Supplementary file 1 — Appendix S1. Practice pattern survey questions (2018 Google Forms), clarification, and limitations. [file ACM2-21-10-s002.docx]

## **Appendix 1: Practice pattern survey questions (2018 Google Forms), clarification, and limitations**

***Part 1: Clarifications and limitations***

There were limits in the survey design which could benefit from commentary or clarification. In general, the definitions of terms used in the survey were based on the AAPM TG132 report^4^ with multi-modality treatment planning (MMTP) defined to be based on image registration of different imaging modalities (e.g. MR, PET, SPECT, CT). In the *standard survey* question: (i) the query on “department responsibilities” did not specify details on what responsibilities related to (e.g. provision of services or assistance); (iii) adoption of DIR did not model rates of decommissioning; (iii) analysis of DIR of dose was grouped for any use case such as for accounting for retreatments or adaptive radiotherapy; the survey on training (vii) did not have a question on a training program for Dosimetrist (an omission by the authors).

For the *extended survey*, departments were surveyed on whether they could rate the level of uncertainty in DIR with images and dose by anatomical site (xii) from a scale of 1 to 5 (low level of uncertainty scaled to 0%, to highest level of uncertainty scaled to 100%); respondents could indicate a non-response if they were unsure (N/A) for uncertainty. For the responses on process based evaluation of (xiii) responsibilities, responses scored which staff groups were involved (multiple responses, or none), and were analysed in terms of average number of staff groups, with a value of less than 1 when no staff groups were involved on average. For process based evaluation of satisfaction levels (xiv), respondents scored from 1 (very dissatisfied) to 3 (neither), to 5 (very satisfied), which was then scaled such that a value of less than 1 indicated unsatisfactory process (scaling of 1 to 0, 3 to 1, 5 to 2); respondents could indicate a non-response if they were unsure (N/A) for process based evaluation. The question on datasets used for validation (xvi) involved checkboxes to denote whether datasets were used by image modality (CT, MR, PET, CBCT, US, 4DCT, 4DCBCT), by phantom type (digital/physical, deformable/rigid), and for clinical data whether it was retrospective or prospective. The question of benefit to risk balance (xxi) was directed towards DIR usage with respondents scoring based on their experience from 1 (risks strongly outweighed benefits) to 3 (risks and benefits similar), to 5 (benefits strongly outweigh risks). These were then scaled such that 1 was 0%, 3 was 50%, and 5 was 100%. On the question of (xxii) quality measures for safe use of RIR/DIR, all responses were open text and analysed descriptively.

***Part 2: Survey questions***

**DATA S1: Per-Department Survey: Practice Pattern for Image Registration Use in WORLD-V3.8**

FINAL EDITION (Anonymised)

Last edit 2018-04-13 9:00AM

* Required

PURPOSE

To gather data on the practice pattern of rigid and deformable image registration use in centres around the world -with respect to the current practice and comparison to AAPM TG132. This data will help in getting a picture of how image registration is used.

WHO TO FILL

1. It is recommended that one survey be filled per department
2. Recommended representatives from Radiation Oncologist/Dosimetrist/Therapist/Physicist team who are responsible for image registration.
3. In practice, it is satisfactory if 1 Physicist and 1 Therapist/Dosimetrist fills it in for the department.

RECOMMENDED TIME:

1) SURVEY TIME: 10 minute for to fill the survey (discussion between parties may increase time) 2) OPTIONAL: For those interested in implementation of deformable image registration, additional questions are available. Please allow 30 minutes to fill this in.

REFERENCE

Brock, K. K., S. Mutic, T. R. McNutt, H. Li and M. L. Kessler (2017). "Use of Image Registration and Fusion Algorithms and Techniques in Radiotherapy: Report of the AAPM Radiation Therapy Committee Task Group No. 132." Med Phys.

**GENERAL FORM INFORMATION**

1. Google forms requires forms does not have a save function for the user. Ideally the user finishes the survey in one sitting or leaves the browser open until finished.
2. It may be helpful to access the PDF of the form for reference before filling in the form (see link)
3. Note that the user can move back and forth between different pages by using the controls in the bottom [BACK][NEXT]

For departments with multiple sites: recommend one survey per site if possible

Otherwise, fill out the as a single department with multiple sites

PRIVACY

-All information is confidential

-Departments have the OPTION to supply email address for further collaboration (otherwise, results are anonymous)

**About your department**

| **Question number** | **Survey questions** | **Respondent answer** |
| --- | --- | --- |
| 1 | Details about your department -public or private | Multiple choice (single selection)  Public, Private, or other |
| 2 | Details about your department -sites per department | Multiple choice (single selection)  Single-site department, Multi-site department, or other |
| 3 | Details about your department -responsibilities | Multiple choice (multiple selections)   External beam radiotherapy  Brachytherapy  Nuclear Medicine  Radiology  University department affiliation  Other |
| 4 | Where is your department | Multiple choice (single selection)  Americas  Europe  Asia  Europe  Oceania  Africa  Other: |
| 5 | How many linac-equivalent treatment delivery systems do you have? | Numeric value  Count treatment delivery systems such as Brachytherapy and Tomotherapy as a linac. |

**What is image registration used for?**

**Questions will be separated into RIGID IMAGE REGISTRATION (RIR) and DEFORMABLE IMAGE REGISTRATION (DIR).**

*DEFINITION: Multi-modality Image Registration*

*Image registration to combine information obtained from different imaging modalities -e.g. MR, PET, SPECT, and CT.*

| **Question number** | **Survey questions** | **Respondent answer** |
| --- | --- | --- |
| 6 | What image modality combinations are used clinically for treatment planning? | RIR: rigid image registration, DIR deformable image registration. Note which combination of DIR/RIR are used in terms of end use (visualisation for target delineation) Check all that apply.  N/A  RIR  DIR  CT-CT  CT-MR  CT-PET  CT-CBCT  CT-US  MR-MR |
| 7 | Other image modality combinations used clinically? | Text response  Please note other image modality combination used. Also note the type of image registration -RIR: rigid image registration, DIR deformable image registration. |

**What is your departments' exposure to deformable image registration software?**

| **Question number** | **Survey questions** | **Respondent answer** |
| --- | --- | --- |
| 8 | DIR SOFTWARE: Which of these deformable image registration software systems has your department had exposure to? | Tick all that apply (tick none if your department has no exposure to deformable image registration software) Check all that apply.  In 2013 (past)  In 2018 (current)  In 2023 (future)  VELOCITY  MIM  MIRADA  PINNACLE with Dynamic planning  License  RAYSTATION with rayDeformable  ADAPTIVO  IMSIMQA  Research software with  deformable image registration |
| 9 | OTHER systems with deformable image registration | Text response  Please include additional information (such as which research packages used, e.g. Plastimatch, ITK, and Slicer) |

| **Question number** | **Survey questions** | **Respondent answer** |
| --- | --- | --- |
| 10 | ATLAS-BASED SEGMENTATION: Are you using Atlas-based segmentation clinically? When did you start (or plan to start)? | Multiple choice (single selection)  Not clinical, no current plans to implement  2021-2026(planned implementation)  2019-2020 (planned implementation)  2018 (implemented, implementing, or planned implementation)  2017  2016  2015  2014  2013  2012  2011  2010  2009  Before 2008 Other: |

| **Question number** | **Survey questions** | **Respondent answer** |
| --- | --- | --- |
| 11 | MULTI-MODAL WITH DEFORMABLE: Are you using deformable image registration for multimodality treatment planning (e.g. for target delineation with PET or MR)? When did you start (or plan to start)? | Multiple choice (single selection)  Not clinical, no current plans to implement  2021-2026(planned implementation)  2019-2020 (planned implementation)  2018 (implemented, implementing, or planned implementation)  2017  2016  2015  2014  2013  2012  2011  2010  2009  Before 2008 Other: |

| **Question number** | **Survey questions** | **Respondent answer** |
| --- | --- | --- |
| 12 | PLAN DOSE DEFORMATION: Are you using deformable image registration to account for retreatment, visualise dose for response assessment, or dose accumulation (e.g. to visualize and avoid overlaps of current and previous dose distribution)? When did you start (or plan to start)? | Multiple choice (single selection)  Not clinical, no current plans to implement  2021-2026(planned implementation)  2019-2020 (planned implementation)  2018 (implemented, implementing, or planned implementation)  2017  2016  2015  2014  2013  2012  2011  2010  2009  Before 2008 Other: |
| 13 | Comments |  |

**How is image registration performed and checked**

| **Question number** | **Survey questions** | **Respondent answer** |
| --- | --- | --- |
| 14 | Has your department adopted the AAPM TG132 recommendations for the TG132 request and report form? | Please refer specifically to implementation of patient specific image registration QA forms (TG132 report form) Mark only one oval.  No, not aware of the report  No, but aware of the report  Yes, currently reviewing and planning implementation  Yes, but do not intend to apply recommendations in the report  Yes, following most of the recommendations of the report  Yes, following all of the recommendations of the report  Other: |
| 15 | What QA mechanisms are currently in place for rigid and deformable image registration for multi-modal treatment planning fusion? | Note all questions are for BOTH rigid and deformable image registration unless specified. Questions for deformable image registration are prefaced by [FOR DIR ONLY] Check all that apply.  N/A (none of the below)  Formal QA check task in system  Registration instructions in protocols (registration landmark, technique, tolerances, etc.)  Registration instructions prescribed by RO (registration landmark, technique, tolerances, etc.)  [FOR DIR ONLY] Qualitative or quantitative checks for deformable vector field and/or deformed image  Registration QA form with achieved accuracy level documented  Decision tree (or equivalent) to perform different actions based on achieved accuracy level (such as re-imaging, increasing margins, side by side only instead of overlay view)  Other: |

| **Question number** | **Survey questions** | **Respondent answer** |
| --- | --- | --- |
| 16 | How is training for rigid (and deformable) image registration currently managed? | Tick all that apply. Comments can be entered in the "other" section (e.g. on any items not covered in checkboxes)  Check all that apply.  Self-training with reading vendor material (e.g. manuals)  Self-training with online material (e.g. video guides)  Self-training with reading/commenting on current standard operating procedures (SOPs)  Informal peer training (e.g. one on one help)  Training from vendor/manufacturer  Competency based assessment (CBA) self-assessed checklist  Competency based assessment (CBA) trainer-assessed checklist  Competency based assessment(CBA) written examination  Competency based assessment(CBA) practical examination  Clinical Training Guide(CTG) or other external training program for registrars/trainees  Training program for Radiation Oncologists  Training program for Radiation Therapists  Training program for Medical Physicists  Anatomical site specific training (e.g. for recognizing landmarks or contouring accuracy)  No formal training program  Other: |

*Clarification for "Estimate the involvement of staff in your department in terms of image registration": guidelines*

1. *Definition of "actively involved"*

*"Actively involved" corresponds to being involved in the use of software -to adjust, QA, and interpret the registration. This relates to active responsibilities which contribution to implementation and operation.*

1. *Consider a department with 10 Radiation Oncologists.*

*If approximately 7 ROs are actively involved in RIR, then choose 60-80% in the next question; If approximately 2 ROs are actively involved in DIR, then choose 0-20% in the next question;*

| **Question number** | **Survey questions** | **Respondent answer** |
| --- | --- | --- |
| 17 | Optional: Your departments definition of "actively involved" | Text response  Each department could potentially have different definitions of what "actively involved" means and what responsibilities are -please describe briefly how each group (RO, RT, ROMP) is "actively involved" in image registration in your department. |
| 18 | Estimate the involvement of staff in your department in terms of image registration | Check approximate staff levels per row. RO refers to Radiation Oncologist, RT refers to Radiation  Therapists, Physics refers to Radiation Oncology Medical Physicists (or Nuclear Medicine Physicists or Diagnostic Imaging Medical Physicists). DOS refer to Dosimetrists. RIR refers to rigid image registration. DIR refers to deformable image registration.  Mark only one oval per row.  0-20  %  20-40  %  40-60  %  60-80  %  %  80-100  RO actively involved in using RIR  RO actively involved in using DIR  RT actively involved in using RIR  RT actively involved in using DIR  Physics actively involved in using  RIR  Physics actively involved in using  DIR  DOS actively involved in using  RIR  DOS actively involved in using  DIR |

| **Question number** | **Survey questions** | **Respondent answer** |
| --- | --- | --- |
| 19 | What are key challenges that your department has found with image registration? | Multiple choice (multiple responses)  Image quality issues (resolution, contrast, orientation, etc.)  Image cropped (scan length, field of view, etc.)  Image transfer (import/export) between multiple systems  Image storage, backup, archive, restore, and infrastructure/networking  Imaging and software accessibility (difficult to use, insufficient licenses, etc.)  Selecting the appropriate image for registration (e.g. selecting correct image(s) from 8+ MR images)  Communication (written or verbal) on required intended use and registration technique  Determining which registration landmark is required  Determining when a registration is satisfactory  Determining what to do when registration is not satisfactory  Documentation of registration accuracy with appropriate follow up  Insufficient training and/or trained staff availability  Insufficient definition of roles and responsibilities  In-house software engineering (such as scripts or extensions) or use of advanced vendor software functions  [DEFORMABLE ONLY] Determining qualitative methods of ensuring deformation is OK  [DEFORMABLE ONLY] Determining quantitative methods of ensuring deformation is OK  Other: |

| **Question number** | **Survey questions** | **Respondent answer** |
| --- | --- | --- |
| 20 | Describe key uncertainties or challenges your department has found with *RIGID* image registration | Text response  Please describe key uncertainties/challenges encountered in your department -if you have had any successful approaches to key challenges, please describe these as well. |
| 21 | Describe key uncertainties or challenges your department has found with *DEFORMABLE* image registration | Text response  Please describe key uncertainties/challenges encountered in your department -if you have had any successful approaches to key challenges, please describe these as well. |

| **Question number** | **Survey questions** | **Respondent answer** |
| --- | --- | --- |
| 22 | Which staff groups have substantial involvement with image registration in your department? | (1) Processes before image registration refer to steps such as ensuring image quality, patient setup, patient preparation, correct image, correct landmarks, etc. (2) Processes involves with image registration and QA refer to steps such as manual registration, setting volume of interest regions for automatic registration, ensuring image registration is reasonable, quality assurance of registration, etc. (3) Processes after image registration refer to communicating the image registration accuracy level, decision process after accuracy level (re-image, margins, etc.), and the end step(target delineation, dose accumulation, etc.). (4) Processes relating to image registration management refers to ensuring roles/responsibilities defined, training, allocation of financial and human resources, risk management, reactive incident monitoring, etc. (5) Decision to clinically release image registration functionality refers to finding a balance of all criteria and ensuring clinical release occurs with a satisfactory balance of benefits to risks Check all that apply.   \|  \| RT \| RO \| Physics \| DOS \| \| --- \| --- \| --- \| --- \| --- \| \| (1) Processes before image registration \| 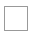 \| 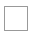 \| 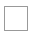 \| 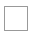 \| \| (2) Processes after image registration \| 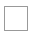 \| 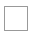 \| 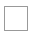 \| 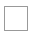 \| \| (3) Processes relating to image registration management and infrastructure \| 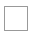 \| 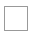 \| 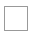 \| 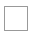 \| \| (4) Decision to clinically release image registration functionality \| 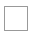 \| 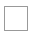 \| 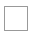 \| 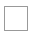 \| |
| 23 | Comment |  |

**Feedback and contact details**

| **Question number** | **Survey questions** | **Respondent answer** |
| --- | --- | --- |
| 24 | What would your department benefit most from in an image registration workshop*? | Text response |
| 25 | Optional: Are there any projects that your department is involved with which could benefit from multi-institutional studies? | Text response |
| 26 | Optional: Enter an email address for further communication (such as collaboration) | Text response |
| 27 | Are you able and willing to participate in more questions? | Text response  Particularly recommended for departments implementing or planning to implement deformable image registration; also for departments implementing or planning to implement AAPM TG132. Your results could be useful for your own department and collated survey results will be made published in the future. |

**EXTENDED SURVEY QUESTIONS**

**EXTENDED QUESTIONS: IDEAL STAFF NUMBERS FOR DEFORMABLE IMAGE REGISTRATION**

| **Question number** | **Survey questions** | **Respondent answer** |
| --- | --- | --- |
| 28 | Ideally, if you had sufficient time and resources to train all staff, estimate what you would want in terms of involvement of staff in terms of deformable image registration | Check approximate staff levels per row. RO refers to Radiation Oncologist, RT refers to Radiation  Therapists, Physics refers to Radiation Oncology Medical Physicists (or Nuclear Medicine Physicists or Diagnostic Imaging Medical Physicists). RIR refers to rigid image registration. DIR refers to deformable image registration.  Mark only one oval per row.  0-20  %  20-40  %  40-60  %  60-80  %  80-100  %  RO actively involved in using DIR  RT actively involved in using DIR  Physics actively involved in using  DIR  DOS actively involved in using  DIR |
| 29 | Comment on staff numbers required for RIR and DIR | Text response |

**EXTENDED QUESTIONS: USE OF DEFORMABLE IMAGE REGISTRATION FOR MULTI-MODALITY TREATMENT PLANNING**

| **Question number** | **Survey questions** | **Respondent answer** |
| --- | --- | --- |
| 30 | MULTI-MODAL TREATMENT PLANNING: What anatomical sites is rigid and/or deformable image registration used clinically? | RIR: rigid image registration, DIR deformable image registration. Note which combination of DIR/RIR are used in terms of end use (visualisation for target delineation) Check all that apply.  Don't know  N/A  RIR  DIR  Brain  Head and neck  Breast  Lung  Esophagus  Pelvis  Prostate  Upper GI  Sarcoma  Haematological |
| 31 | Comment |  |

**EXTENDED QUESTIONS: USE OF DEFORMABLE IMAGE REGISTRATION FOR ACCOUNTING FOR RETREATMENT**

| **Question number** | **Survey questions** | **Respondent answer** |
| --- | --- | --- |
| 32 | ACCOUNTING FOR RE-TREATMENT: What anatomical sites is rigid and/or deformable image registration used clinically? | RIR: rigid image registration, DIR deformable image registration.  Check all that apply.  Don't know  N/A  RIR  DIR  Brain  Head and neck  Breast  Lung  Esophagus  Pelvis  Prostate  Upper GI  Sarcoma  Haematological |
| 33 | Comment |  |

EXTENDED QUESTIONS: USE OF DEFORMABLE IMAGE

REGISTRATION FOR ACCOUNTING FOR ADAPTIVE RETREATMENT

| **Question number** | **Survey questions** | **Respondent answer** |
| --- | --- | --- |
| 34 | ADAPTIVE RADIOTHERAPY: What anatomical sites is rigid and/or deformable image registration used clinically? | RIR: rigid image registration, DIR deformable image registration.  Check all that apply.  Don't know  N/A  RIR  DIR  Brain  Head and neck  Breast  Lung  Esophagus  Pelvis  Prostate  Upper GI  Sarcoma  Haematological |
| 35 | Comment |  |

**EXTENDED QUESTIONS: USE OF DEFORMABLE IMAGE REGISTRATION FOR ATLAS BASED SEGMENTATION**

| **Question number** | **Survey questions** | **Respondent answer** |
| --- | --- | --- |
| 36 | ATLAS BASED SEGMENTATION: What anatomical sites are used clinically in terms of manual contouring vs. atlas based segmentation? * | Where editing refers to manual methods of contouring and editing contours based on human operator. Where auto refers to atlas based segmentation or other automated contouring methods. Choose the option that best represents what is being done for each site.  Mark only one oval per row.  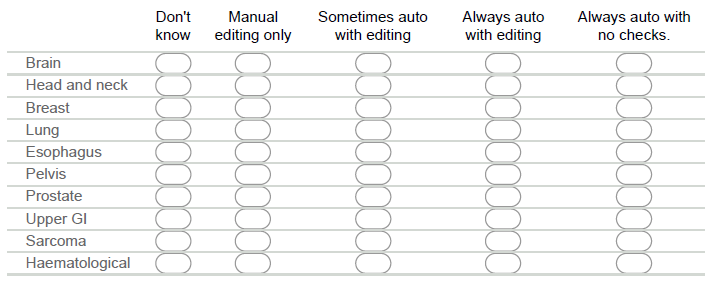 |
| 37 | Comment |  |

**EXTENDED QUESTIONS: PROCESSES BEFORE IMAGE**

**REGISTRATION [UPSTREAM]**

| **Question number** | **Survey questions** | **Respondent answer** |
| --- | --- | --- |
| 38 | ROLES AND RESPONSIBILITIES [UPSTREAM]: Who is/are involved with the following processes? | Tick all that apply regarding staff group(s) involved - multiple groups can be ticked. If there are no staff allocated to a particular process, tick N/A. (if there are a range of answers, multiple selections is possible. e.g. for some sites, implicit registration prescription is performed always and for other sites it is not performed.). For answers not contained in the options, fill in a brief description in the next section  (comment section)  Check all that apply.   \|  \| N/A \| RTs \| ROs \| Physics \| DOS \| \| --- \| --- \| --- \| --- \| --- \| --- \| \| Processes to ensure sufficient information in image (scan length and FOV sufficient to avoid missing information) \| 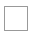 \| 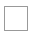 \| 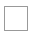 \| 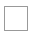 \| 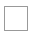 \| \| Processes to ensure Setup factors between images optimised (e.g. immobilisation and setup) \| 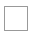 \| 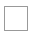 \| 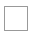 \| 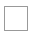 \| 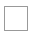 \| \| Processes to ensure patient factors between scans optimised (e.g. patient preparation such as bowel prep) \| 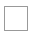 \| 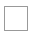 \| 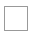 \| 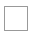 \| 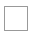 \| \| Processes to ensure image quality optimised (e.g. scan protocol, noise, resolution, artefacts) \| 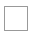 \| 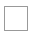 \| 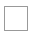 \| 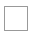 \| 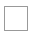 \| \| Processes for satisfactory orientation and data integrity \| 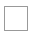 \| 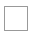 \| 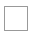 \| 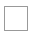 \| 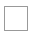 \| \| Processes to ensure correct image registered (e.g. wrong MRN, date or series, right image registered) \| 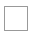 \| 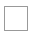 \| 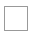 \| 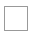 \| 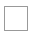 \| \| Processes to validate incorporation of previous RT information (e.g. ensuring correct dose prescription, scaling of doses, correct contour structures, etc.) \| 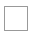 \| 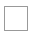 \| 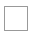 \| 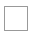 \| 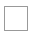 \| \| Processes for Implicit registration prescription (e.g. protocol defining what landmark to register to, what registration technique, tolerances, and action thresholds) \| 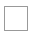 \| 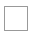 \| 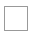 \| 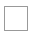 \| 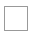 \| \| Processes to prepare explicit registration prescription (e.g. RO writing patient specific instructions on what landmark to register to, what registration technique, tolerances, and action thresholds) \| 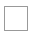 \| 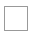 \| 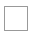 \| 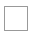 \| 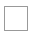 \| |

| **Question number** |  |
| --- | --- |
| 39 | EVALUATION [UPSTREAM]: How satisfied are you with these processes in your department? * |
| **Survey questions** | |
| Please describe how satisfied your department is in terms of the following processes (note- you could potentially consider how often issues occur, how severe issues are when they occur, or how often issues are detected with current quality control systems) Rating: N/A-not existent, 1-Very dissatisfied, 2Dissatisfied, 3-Neither satisfied or dissatisfied, 4-Satisfied, 5-Very satisfied Mark only one oval per row.   \|  \| N/A \| Very dissatisfied \| Dissatisfied \| Neither satisfied or dissatisfied \| Satisfied \| Very satisfied \| \| --- \| --- \| --- \| --- \| --- \| --- \| --- \| \| Processes to ensure sufficient information in image (scan length and FOV sufficient to avoid missing information) \| 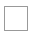 \| 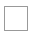 \| 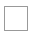 \| 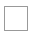 \| 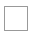 \| 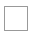 \| \| Processes to ensure Setup factors between images optimised (e.g. immobilisation and setup) \| 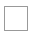 \| 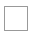 \| 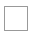 \| 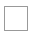 \| 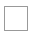 \| 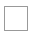 \| \| Processes to ensure patient factors between scans optimised (e.g. patient preparation such as bowel prep) \| 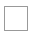 \| 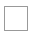 \| 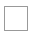 \| 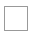 \| 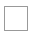 \| 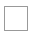 \| \| Processes to ensure image quality optimised (e.g. scan protocol, noise, resolution, artefacts) \| 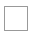 \| 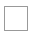 \| 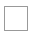 \| 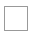 \| 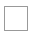 \| 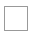 \| \| Processes for satisfactory orientation and data integrity \| 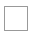 \| 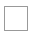 \| 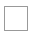 \| 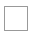 \| 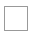 \| 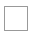 \| \| Processes to ensure correct image registered (e.g. wrong MRN, date or series, right image registered) \| 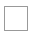 \| 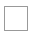 \| 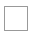 \| 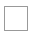 \| 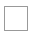 \| 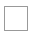 \| \| Processes to validate incorporation of previous RT information (e.g. ensuring correct dose prescription, scaling of doses, correct contour structures, etc.) \| 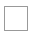 \| 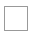 \|  \|  \|  \|  \| \| Processes for Implicit registration prescription (e.g. protocol defining what landmark to register to, what registration technique, tolerances, and action thresholds) \|  \|  \|  \|  \|  \|  \| \| Processes to prepare explicit registration prescription (e.g. RO writing patient specific instructions on what landmark to register to, what registration technique, tolerances, and action thresholds) \|  \|  \|  \|  \|  \|  \| | |

| **Question number** | **Survey questions** | **Respondent answer** |
| --- | --- | --- |
| 40 | Comment |  |

**EXTENDED QUESTIONS: PROCESSES INVOLVING IMAGE**

**REGISTRATION [REGISTRATION]**

| **Question number** | **Survey questions** | **Respondent answer** |
| --- | --- | --- |
| 41 | ROLES AND RESPONSIBILITIES [REGISTRATION]: Who is/are involved with the following processes? | Tick all that apply regarding staff group(s) involved - multiple groups can be ticked. If there are no staff allocated to a particular process, tick N/A. (if there are a range of answers, multiple selections is possible. e.g. for some sites, implicit registration prescription is performed always and for other sites it is not performed.). For answers not contained in the options, fill in a brief description in the next section  (comment section)  Check all that apply.   \|  \| N/A \| RTs \| ROs \| Physics \| DOS \| \| --- \| --- \| --- \| --- \| --- \| --- \| \| Processes for interpreting implicit/explicit registration prescription (landmarks/local region, thresholds, etc.) \|  \|  \|  \|  \|  \| \| Registration technique optimal (rigid) \|  \|  \|  \|  \|  \| \| Registration technique optimal (deformable) \|  \|  \|  \|  \|  \| \| Processes to ensure image quality optimised (e.g. scan protocol, noise, resolution, artefacts) \|  \|  \|  \|  \|  \| \| Landmarks in image identified (e.g. staff knowledge of anatomy/GTV) \|  \|  \|  \|  \|  \| \| Registration QA (qualitative and/or quantitative, checked with thresholds, and user understanding) \|  \|  \|  \|  \|  \| \| Uncertainty/issues documented (e.g. areas of deformation, artefacts, etc.) \|  \|  \|  \|  \|  \| \| Accuracy level documented and reported \|  \|  \|  \|  \|  \| |

| **Question number** |  |
| --- | --- |
| 42 | EVALUATION [REGISTRATION]: How satisfied are you with these processes in your department? |
| **Survey questions** | |
| Please describe how satisfied your department is in terms of the following processes (note- you could potentially consider how often issues occur, how severe issues are when they occur, or how often issues are detected with current quality control systems) Rating: N/A-not existent, 1-Very dissatisfied, 2Dissatisfied, 3-Neither satisfied or dissatisfied, 4-Satisfied, 5-Very satisfied Mark only one oval per row.   \|  \| N/A \| Very dissatisfied \| Dissatisfied \| Neither satisfied or dissatisfied \| Satisfied \| Very satisfied \| \| --- \| --- \| --- \| --- \| --- \| --- \| --- \| \| Processes for interpreting implicit/explicit registration prescription (landmarks/local region, thresholds, etc.) \|  \|  \|  \|  \|  \|  \| \| Registration technique optimal (rigid) \|  \|  \|  \|  \|  \|  \| \| Registration technique optimal (deformable) \|  \|  \|  \|  \|  \|  \| \| Processes to ensure image quality optimised (e.g. scan protocol, noise, resolution, artefacts) \|  \|  \|  \|  \|  \|  \| \| Landmarks in image identified (e.g. staff knowledge of anatomy/GTV) \|  \|  \|  \|  \|  \|  \| \| Registration QA (qualitative and/or quantitative, checked with thresholds, and user understanding) \|  \|  \|  \|  \|  \|  \| \| Uncertainty/issues documented (e.g. areas of deformation, artefacts, etc.) \|  \|  \|  \|  \|  \|  \| \| Accuracy level documented and reported \|  \|  \|  \|  \|  \|  \| | |

| **Question number** | **Survey questions** | **Respondent answer** |
| --- | --- | --- |
| 43 | Comment |  |

**EXTENDED QUESTIONS: PROCESSES AFTER IMAGE**

**REGISTRATION [DOWNSTREAM]**

| **Question number** | **Survey questions** | **Respondent answer** |
| --- | --- | --- |
| 44 | ROLES AND RESPONSIBILITIES [DOWNSTREAM]: Who is/are involved with the following processes? | Tick all that apply regarding staff group(s) involved - multiple groups can be ticked. If there are no staff allocated to a particular process, tick N/A. (if there are a range of answers, multiple selections is possible. e.g. for some sites, implicit registration prescription is performed always and for other sites it is not performed.). For answers not contained in the options, fill in a brief description in the next section  (comment section)  Check all that apply.   \|  \| N/A \| RTs \| ROs \| Physics \| DOS \| \| --- \| --- \| --- \| --- \| --- \| --- \| \| Registration results interpreted (TG132 report) \|  \|  \|  \|  \|  \| \| Decision when whole scan [level 0] or local regions [level 1] are aligned \|  \|  \|  \|  \|  \| \| Decision when usable with risk of deformation [level 2] (additional PTV margin may be required as per TG 132) \|  \|  \|  \|  \|  \| \| Processes to calculate and apply margin policy (accuracy level 2) \|  \|  \|  \|  \|  \| \| Process followed when image registration is usable for diagnosis only [level 3] or not for clinical use [level 4] \|  \|  \|  \|  \|  \| \| Process to ensure atlas based contours checked/edited/finalised \|  \|  \|  \|  \|  \| \| Process to process/validate deformed image and dose (e.g. QA, BED calcs, scaling, etc.) \|  \|  \|  \|  \|  \| \| Process to document registration QA and actions in hospital database (e.g. TG132 report form) \|  \|  \|  \|  \|  \| |

| **Question number** |  |
| --- | --- |
| 45 | EVALUATION [DOWNSTREAM]: How satisfied are you with these processes in your department? |
| **Survey questions** | |
| Tick all that apply regarding staff group(s) involved - multiple groups can be ticked. If there are no staff allocated to a particular process, tick N/A. (if there are a range of answers, multiple selections is possible. e.g. for some sites, implicit registration prescription is performed always and for other sites it is not performed.). For answers not contained in the options, fill in a brief description in the next section   \|  \| N/A \| Very dissatisfied \| Dissatisfied \| Neither satisfied or dissatisfied \| Satisfied \| Very satisfied \| \| --- \| --- \| --- \| --- \| --- \| --- \| --- \| \| Registration results interpreted (TG132 report) \|  \|  \|  \|  \|  \|  \| \| Decision when whole scan [level 0] or local regions [level 1] are aligned \|  \|  \|  \|  \|  \|  \| \| Decision when usable with risk of deformation [level 2] (additional PTV margin may be required as per TG 132) \|  \|  \|  \|  \|  \|  \| \| Processes to calculate and apply margin policy (accuracy level 2) \|  \|  \|  \|  \|  \|  \| \| Process followed when image registration is usable for diagnosis only [level 3] or not for clinical use [level 4] \|  \|  \|  \|  \|  \|  \| \| Process to ensure atlas based contours checked/edited/finalised \|  \|  \|  \|  \|  \|  \| \| Process to process/validate deformed image and dose (e.g. QA, BED calcs, scaling, etc.) \|  \|  \|  \|  \|  \|  \| \| Process to document registration QA and actions in hospital database (e.g. TG132 report form) \|  \|  \|  \|  \|  \|  \| | |

| **Question number** | **Survey questions** | **Respondent answer** |
| --- | --- | --- |
| 46 | Comment |  |

**EXTENDED QUESTIONS: MANAGEMENT OF IMAGE**

**REGISTRATION [MANAGEMENT]**

| **Question number** | **Survey questions** | **Respondent answer** |
| --- | --- | --- |
| 47 | ROLES AND RESPONSIBILITIES [MANAGEMENT]: Who is/are involved with the following processes? | Tick all that apply regarding staff group(s) involved - multiple groups can be ticked. If there are no staff allocated to a particular process, tick N/A. (if there are a range of answers, multiple selections is possible. e.g. for some sites, implicit registration prescription is performed always and for other sites it is not performed.). For answers not contained in the options, fill in a brief description in the next section  (comment section)  Check all that apply.   \|  \| N/A \| RTs \| ROs \| Physics \| DOS \| \| --- \| --- \| --- \| --- \| --- \| --- \| \| Managing roles/responsibilities with allocating time from trained staff to known task times \|  \|  \|  \|  \|  \| \| Quality management of imaging equipment (e.g. CTs) and infrastructure (e.g. PACS) \|  \|  \|  \|  \|  \| \| Sufficient datasets, validation, and procedures in place (commissioning and operation) \|  \|  \|  \|  \|  \| \| Coordination and integration with RO, RT, Physics as well as various portfolios (planning, imaging, treatment, etc.). Also with Nuclear Medicine, Radiology, Medical Oncology, etc. \|  \|  \|  \|  \|  \| \| In-house software engineering or use of advanced vendor functions \|  \|  \|  \|  \|  \| \| Project management: balancing quality, risk, and efficiency \|  \|  \|  \|  \|  \| \| Reactive systems: all technical and human issues, incidents and near misses are managed \|  \|  \|  \|  \|  \| |

| **Question number** |  |
| --- | --- |
| 48 | EVALUATION [MANAGEMENT]: How satisfied are you with these processes in your department? |
| **Survey questions** | |
| Tick all that apply regarding staff group(s) involved - multiple groups can be ticked. If there are no staff allocated to a particular process, tick N/A. (if there are a range of answers, multiple selections is possible. e.g. for some sites, implicit registration prescription is performed always and for other sites it is not performed.). For answers not contained in the options, fill in a brief description in the next section  (comment section)  Mark only one oval per row.   \|  \| N/A \| Very dissatisfied \| Dissatisfied \| Neither satisfied or dissatisfied \| Satisfied \| Very satisfied \| \| --- \| --- \| --- \| --- \| --- \| --- \| --- \| \| Managing roles/responsibilities with allocating time from trained staff to known task times \|  \|  \|  \|  \|  \|  \| \| Quality management of imaging equipment (e.g. CTs) and infrastructure (e.g. PACS) \|  \|  \|  \|  \|  \|  \| \| Sufficient datasets, validation, and procedures in place (commissioning and operation) \|  \|  \|  \|  \|  \|  \| \| Coordination and integration with RO, RT, Physics as well as various portfolios (planning, imaging, treatment, etc.). Also with Nuclear Medicine, Radiology, Medical Oncology, etc. \|  \|  \|  \|  \|  \|  \| \| In-house software engineering or use of advanced vendor functions \|  \|  \|  \|  \|  \|  \| \| Project management: balancing quality, risk, and efficiency \|  \|  \|  \|  \|  \|  \| \| Reactive systems: all technical and human issues, incidents and near misses are managed \|  \|  \|  \|  \|  \|  \| | |

| **Question number** | **Survey questions** | **Respondent answer** |
| --- | --- | --- |
| 49 | Comment |  |

**EXTENDED QUESTIONS: REGISTRATION VALIDATION METRICS**

**AND DATASETS [VALIDATION]**

| **Question number** |  |
| --- | --- |
| 50 | Which per-patient Registration QA metrics are in use, and who assesses them? |
| **Survey questions** | |
| See Fig 5. In AAPM TG132 for reference. Check all that apply.   \|  \| N/A \| RTs \| ROs \| Physics \| DOS \| \| --- \| --- \| --- \| --- \| --- \| --- \| \| Qualitative Metrics: anatomical landmarks visualised (spyglass, checker box, etc.) \|  \|  \|  \|  \|  \| \| Qualitative Metrics: anatomical landmarks captured with screenshots \|  \|  \|  \|  \|  \| \| Qualitative Metrics: anatomical landmarks with grid/ruler in software \|  \|  \|  \|  \|  \| \| Qualitative Metrics: comparison with contours(with expansion/contractions) \|  \|  \|  \|  \|  \| \| Quantitative Metrics: target registration error(TRE) \|  \|  \|  \|  \|  \| \| Quantitative Metrics: mean distance to agreement(MDA) \|  \|  \|  \|  \|  \| \| Quantitative Metrics: dice similarity coefficient(DSC) \|  \|  \|  \|  \|  \| \| Quantitative Metrics: jacobian(<1 vol-expand, 0-1 vol-reduced, 1-nochange, <0 erroneous) \|  \|  \|  \|  \|  \| \| Quantitative Metrics: consistency(transitivity A-B and B-C vs. A-C) \|  \|  \|  \|  \|  \| \| Subjective considerations (e.g. tumour growth, patient changes noted) \|  \|  \|  \|  \|  \| | |

| **Question number** | **Survey questions** | **Respondent answer** |
| --- | --- | --- |
| 51 | Registration QA validation: which datasets are used for validation? | Check all that apply. |
| 52 | Comment |  |

| **Question number** | **Survey questions** | **Respondent answer** |
| --- | --- | --- |
| 53 | Registration QA validation: number of datasets in your atlas (atlas based segmentation) | Numeric response  *Note the number of datasets you use per atlas |
| 54 | Registration QA validation: number of datasets evaluated before clinical release (atlas based segmentation) | Numeric response  *Note the number of datasets that you have or will use before clinical release –to verify your atlas |
| 55 | Registration QA validation: How often is patient-specific QA performed for atlas based segmentation * | Patient specific QA that will be performed after clinical release Mark only one oval.  1 2 3 4 5  Rarely  Always |
| 56 | Comment |  |
| 57 | Registration QA validation: number of datasets evaluated before clinical release (multi-modality treatment planning image registration) * | Numeric response  *Note the number of datasets that you have or will use before clinical release |
| 58 | Registration QA validation: How often is patient-specific QA performed for multi-modality treatment planning image registration | Patient specific QA that will be performed after clinical release Mark only one oval.  1 2 3 4 5  Rarely  Always |
| 59 | Comment |  |

| **Question number** | **Survey questions** | **Respondent answer** |
| --- | --- | --- |
| 60 | Registration QA validation: number of datasets evaluated before clinical release for image registration related to RT dose (accounting for retreatment, dose response assessment, etc.) | Numeric response  *Note the number of datasets that you have or will use before clinical release |
| 61 | Registration QA validation: How often is patient-specific QA performed for image registration related to RT dose (e.g. accounting for retreatment, dose response assessment) | Patient specific QA that will be performed after clinical release Mark only one oval.  1 2 3 4 5  Rarely  Always |
| 62 | Comment |  |
| 63 | Registration QA validation: number of datasets evaluated before clinical release (adaptive radiotherapy) | Numeric response  *Note the number of datasets that you have or will use before clinical release |
| 64 | Registration QA validation: How often is patient-specific QA performed for image registration related to adaptive radiotherapy | Patient specific QA that will be performed after clinical release Mark only one oval.  1 2 3 4 5  Rarely  Always |
| 65 | Comment |  |

EXTENDED QUESTIONS: NON-STANDARD IMAGE REGISTRATION

TECHNIQUES

| **Question number** | **Survey questions** | **Respondent answer** |
| --- | --- | --- |
| 66 | EXTENDED QUESTIONS: NON-STANDARD IMAGE REGISTRATION TECHNIQUES: Does your department use the following |  |
| 67 | Comment |  |

EXTENDED QUESTIONS: DECISION MAKING AND CRITERIA FOR CLINICAL RELEASE

| **Question number** | **Survey questions** | **Respondent answer** |
| --- | --- | --- |
| 68 | EXTENDED QUESTIONS: DECISION MAKING AND CRITERIA FOR CLINICAL RELEASE, TOP 3 CRITERIA in terms of PRIORITY: In commissioning and implementing image registration, what would your department consider in terms of the highest in terms of priority? | Rationale: The actual implementation may involve a balance between various objectives -some of which may conflict (such as quality vs. cost) --a best solution would depend on the priority in the criteria which could be department specific Check all that apply.   - QUALITY: system to measure and improve quality - EFFECTIVENESS: registration quality and QA optimized (e.g. optimal setup or registration technique with DIR) - FEASIBILITY: tasks practical and achievable - COSTS: ongoing/recurring human (workload) and financial (equipment) - EFFICIENCY: rapid progress and clinical release - DOCUMENTATION: registration quality and error handling (TG132 report) part of medical records - PROACTIVE SYSTEMS: validation and risks managed (e.g. artefacts and uncertainties) - REACTIVE SYSTEMS: per patient/system review and decisions (e.g. uncertainties, risks, etc.) - ROLES/TRAINING: specified and managed - COMPLIANCE: best practice achieved (e.g. TG132) - UNCERTAINTY/RISKS: known, documented, and managed |
| 69 | Comments regarding criteria for clinical release for rigid image registration | Text response |
| 70 | Comments regarding criteria for clinical release for deformable image registration | Text response |

**EXTENDED QUESTIONS: UNCERTAINTIES**

| **Question number** | **Survey questions** | **Respondent answer** |
| --- | --- | --- |
| 71 | What are key uncertainties? | Text response  (1) Describe key uncertainties that are an obstacle to your department adopting DIR clinically. (2) How have you managed to deal with uncertainties in clinical implementation, or (3) are there uncertainties that are still difficult to solve? |
| 72 | Uncertainty by anatomical site: deformable image registration with images and dose | Mark only one oval per row. |

**EXTENDED QUESTIONS: OVERALL BALANCE OF BENEFITS AND**

**RISKS FOR YOUR DEPARTMENT**

| **Question number** | **Survey questions** | **Respondent answer** |
| --- | --- | --- |
| 73 | Based on the data in the literature and in your department, what is the current benefit to risk balance in terms of using DIR functionalities (as opposed to traditional or rigid based techniques)? | In consideration of the OVERALL risks and benefits -based on your department's current state in terms of all factors, e.g. technical quality issues, human resource issues, clinical rationale, financial costs, workload, efficiency, etc. What does your department consider is "worth doing (benefits>risks)" as opposed to needing further work to increasing the level of benefits or decreasing the level of risks (or costs).  Mark only one oval per row.   \|  \| Risks outweigh benefits significantly (further work required) \| Risks outweigh benefits moderately (further work required) \| Risk similar to benefits (further work required)( \| Benefits outweigh risks moderately (clinical use) \| Benefits outweigh risks significantly (clinical use) \| \| --- \| --- \| --- \| --- \| --- \| --- \| \| Atlas based segmentation \|  \|  \|  \|  \|  \| \| Multimodality treatment planning \|  \|  \|  \|  \|  \| \| Accounting for retreatment, response assessment \|  \|  \|  \|  \|  \| \| Adaptive radiotherapy \|  \|  \|  \|  \|  \| |
| 74 | What are the key factors affecting the overall risk-benefit balance (e.g. what makes it not worth doing?) | Text response  In contrast to the question on uncertainties (slanted towards technical factors), include any nontechnical factors such as cost, efficiency, workload, training, etc. |

**EXTENDED QUESTIONS: QUALITY IMPROVEMENT CYCLES AND**

**SAFETY SYSTEM**

These questions relate to Plan-Do-Study-Act (PDSA) quality improvement cycle methodology and also incident reporting management strategies.

| **Question number** | **Survey questions** | **Respondent answer** |
| --- | --- | --- |
| 75 | What measures would you (or have you) use to measure the quality of implementing rigid image registration? E.g. How would you know that the project is successful? | Text responses |
| 76 | What measures would you (or have you) use to measure the safety of implementing rigid image registration? E.g. How would you know if there is a near miss or adverse incident? | Text responses |
| 77 | Comments on quality and safety of rigid image registration | Text responses |
| 78 | What measures would you (or have you) use to measure the quality of implementing deformable image registration? E.g. How would you know that the project is successful? | Text responses |
| 79 | What measures would you (or have you) use to measure the safety of implementing deformable image registration? E.g. How would you know if there is a near miss or adverse incident? | Text responses |
| 80 | Comments on quality and safety of deformable image registration | Text responses |
| 81 | General feedback on survey | Text responses |

Powered by
