# Supplementary material for: An international survey on the clinical use of rigid and deformable image registration in radiotherapy
Source: J Appl Clin Med Phys. 2020 Sep 11;21(10):10–24. doi: 10.1002/acm2.12957 (PMC7075391; doi:10.1002/acm2.12957)
Supplement: Supplementary file 2 — Appendix S2. Open responses to adoption of DIR by use case. [file ACM2-21-10-s001.docx]

## **Appendix 2: Open responses to adoption of DIR by use case**

The following is a summary of the open text comments for the questions relating to adoption of DIR adoption by use case. Responses for atlas based segmentation noted that centres where no atlas based segmentation was used, automatic tools for contouring and processing were employed instead. Some centres indicated that the reason for a lack of uptake was that Oncologists were not satisfied with the accuracy of auto segmentation. Other centres routinely used atlas based segmentation for multiple anatomical sites, for the generation of new plans or for rescan and replans.

For multi-modality treatment planning, centres commented how the Oncologist making the decision of using RIR or DIR communicated to Physicists and Dosimetrists based on the patient's anatomy and scan timing relative to surgery or chemotherapy. Some centres noted that extreme care would be required for DIR especially for soft tissue environments such as the abdomen and particularly in collapsed lung cases. Other centres approached multi-modality imaging with efforts to reduce differences with radiotherapy treatment masks in MR or scanning PET CT with a vacuum bag and a radiotherapy couch top.

In terms of DIR for accounting for retreatment, some centres reported that they only used DIR when RIR seems insufficient, whilst other centres routinely used DIR by anatomical sites (such as H&N retreatments). Some centres used DIR at various levels, such as using DIR to map structures between the datasets in all cases, and where DIR shows excellent agreement and is physically reasonable using DIR for dose deformation.

When using DIR with dose, some centres reported limiting the scope of dose deformation only as a gross visual tool with limited confidence in its accuracy and noted use was not always appropriate based on significant anatomical changes. Some centres prefer use of RIR where possible and only used DIR for retreatment assessment rarely and when absolutely necessary. Some noted the use of multiple DIR systems for registration whilst others commented on informal use of DIR with formalisation of processes prompted by the publication of AAPM TG132. Some used dose deformation from a research capacity, whilst others use it clinically with substantial analysis of the vector field, deformation grid, physical reasonability of deformation, and a stringent reporting system. Obstacles for implementation were cost and human resources for some, whilst others reported preparedness for use of DIR but without RO support.

Respondents noted that DIR for adaptive radiotherapy involved limiting this application to selected anatomical sites (Head and Neck) but not to other sites due to limited verification data. Some centres reported development of high quality CBCT acquisitions suitable for dose calculation of delivered dose on treatment images. Some centres visualised the delivered dose distributions on the treatment images without deforming the dose from the CBCT to the planning CT, but some centres used RIR of the CBCT or rescan CT to account for changes in tumor or patient anatomy during treatment.
